# Distinct contributions of GluA1-containing AMPA receptors of different hippocampal subfields to salience processing, memory and impulse control

**DOI:** 10.1038/s41398-022-01863-8

**Published:** 2022-03-14

**Authors:** Kasyoka Kilonzo, Daniel Strahnen, Vivien Prex, John Gems, Bastiaan van der Veen, Sampath K. T. Kapanaiah, Bhargavi K. B. Murthy, Stefanie Schulz, Rolf Sprengel, David Bannerman, Dennis Kätzel

**Affiliations:** 1 Institute of Applied Physiology, Ulm University, Ulm, Germany; 2 Max Planck Institute for Medical Research, Heidelberg, Germany; 3 Department of Experimental Psychology, University of Oxford, Oxford, UK

**Keywords:** Hippocampus, Molecular neuroscience

## Abstract

Schizophrenia is associated with a broad range of severe and currently pharmacoresistant cognitive deficits. Prior evidence suggests that hypofunction of AMPA-type glutamate receptors (AMPARs) containing the subunit GLUA1, encoded by *GRIA1*, might be causally related to impairments of selective attention and memory in this disorder, at least in some patients. In order to clarify the roles of GluA1 in distinct cell populations, we investigated behavioural consequences of selective *Gria1-*knockout in excitatory neurons of subdivisions of the prefrontal cortex and the hippocampus, assessing sustained attention, impulsivity, cognitive flexibility, anxiety, sociability, hyperactivity, and various forms of short-term memory in mice. We found that virally induced reduction of GluA1 across multiple hippocampal subfields impaired spatial working memory. Transgene-mediated ablation of GluA1 from excitatory cells of CA2 impaired short-term memory for conspecifics and objects. *Gria1* knockout in CA3 pyramidal cells caused mild impairments of object-related and spatial short-term memory, but appeared to partially increase social interaction and sustained attention and to reduce motor impulsivity. Our data suggest that reduced hippocampal GluA1 expression—as seen in some patients with schizophrenia—may be a central cause particularly for several short-term memory deficits. However, as impulse control and sustained attention actually appeared to improve with GluA1 ablation in CA3, strategies of enhancement of AMPAR signalling likely require a fine balance to be therapeutically effective across the broad symptom spectrum of schizophrenia.

## Introduction

AMPA (α-amino-3-hydroxy-5-methyl-4-isoxazolepropionic acid) receptors (AMPARs) containing the GluA1 subunit are essential for synaptic plasticity [1–4] and have been implicated in a variety of processes related to psychiatric diseases. For example, GluA1-containing AMPARs in prefrontal excitatory cells are essential downstream targets mediating the maintenance of the anti-depressant response to ketamine in rodents [5]. The same receptors are also modulated specifically by alpha2-adrenoreceptors, the target of the attention-deficit-hyperactivity disorder drug guanfacine [6]. Most prominently, GLUA1 has been implicated in schizophrenia [7]. *GRIA1*, the gene encoding GLUA1, has been identified as a risk gene for this disease in genome-wide association studies [8–10]. Decreased *GRIA1* mRNA and protein levels have been found in hippocampal tissue from schizophrenia patients in three studies using either in situ hybridisation (ISH) or autoradiography, respectively [11–13]. Although note that three other studies using ISH or Western Blot, respectively, did not find significantly altered *GRIA1* expression in hippocampi from schizophrenia patients [14–16]. This suggests that a hypofunction of GLUA1 may be one out of multiple molecular pathological alterations that can each impair synaptic function in the hippocampus [17, 18] and thereby causally contribute to schizophrenia.

Global *Gria1* ablation in mice has been used to model certain deficits of synaptic plasticity, dopamine regulation and associated psychological functions related to schizophrenia [7, 19, 20]. *Gria1*-knockout (*Gria1*^*−/−*^) mice show severe impairments of spatial short-term memory and of attentional regulation of novelty-related salience attribution to objects, spatial and non-spatial cues [7, 21, 22], while, at the same time, they retain the ability to form spatial long-term memories [23, 24]. GluA1-related failures of salience attribution are likely driven by impaired short-term habituation [7], a psychological mechanism that reduces attention paid to stimuli as they become familiar [7, 25], and whose failure may cause excessive novelty-induced hyperlocomotion and impaired spatial and non-spatial novelty preference in rodents. *Gria1*-knockout also provokes a hyperdopaminergic state in the striatum [26]—indicative of aberrant salience attribution [20, 25, 27]—and defective pre-pulse inhibition [26].

However, the clinical strategy of enhancing AMPAR function indiscriminately with AMPAkines has been met with mixed results [28–30]. In order to evaluate the enhancement of GluA1-AMPAR function as a therapeutic concept in schizophrenia, it is vital to understand through which cell types and neural circuits GluA1 hypofunction may contribute to specific psychiatric deficits and to identify its broader contributions to cognition. However, circuit- or cell-type-specific studies of GluA1-functions were so far limited to assessments of short-term memory in mice with either broader but mosaic restoration of forebrain or hippocampal GluA1 expression in *Gria1*^*−/−*^ mice [31–34] or with Cre-mediated ablation in parvalbumin interneurons [35] or in different hippocampal subdivisions [36]. We have recently identified a specific role of GluA1 in the CA2/CA3 subfields of the hippocampus, as impaired spatial short-term habituation in *Gria1*^*−/−*^ mice could be restored by viral reintroduction of GluA1 into these circuits [33]. It remains to be clarified, however, which cell types and subfields are responsible for this rescue and whether the role of GluA1 in CA2/3 GluA1 extends to other schizophrenia-related phenotypes. For example, it is uncertain which GluA1-containing AMPARs support short-term memory performance across different tasks [31, 33]. To dissect the role of GluA1 in distinct neuronal circuits, here we examined the behavioural consequences of *Gria1* ablation in excitatory cells of either prefrontal cortex or hippocampus in a broad battery of behavioural assays, including back-translational tasks relevant to schizophrenia.

## Methods

### Animals

All experiments were performed in accordance with the German Animal Rights Law (Tierschutzgesetz) 2013 and the European Union regulations for the use of laboratory animals (EU Directive 2010/63), and were approved by the Federal Ethical Review Committee (Regierungspräsidium Tübingen) of Baden-Württemberg, Germany (license number TV1399). To enable region- and excitatory cell-type selective *Gria1*-ablation, mice with a homozygously floxed *Gria1*-locus (*Gria1*^*f/f*^, B6N.129-Gria1^tm2Rsp^/J; also known as GluR-A [2*lox*], Jax stock# 019012) were either infused with a Cre- or a GFP-expressing rAAV-vector (AAV8-CamKIIα-GFP-Cre for Gria1-knockout, AAV8-CamKIIα-GFP for controls; UNC vector core; *AAV-cohort*) at 6 months of age, on average, or were cross-bred with Cre-driver lines in which promoters directed Cre-expression to excitatory cells of either CA2 (*Tg*^*Amigo2-Cre*^, B6.Cg-Tg(Amigo2-cre)1Sieg/J, Jax stock# 030215) [37] or CA3 (Grik4-Cre, C57BL/6-*Tg*(Grik4-cre)G32-4Stl/J, Jax-stock# 006474) [38], *transgenic cohort*. In the latter cases, breedings between *Gria1*^*f/f*^ x *Tg*^*Amigo2-cre*^ and *Gria1*^*f/f*^ x *Tg*^*GriK4-cre*^ genotypes were performed to obtain *Gria1*^*ΔAmigo2*^ and *Gria1*^*ΔGriK4*^ offsprings and their *Gria1*^*f/f*^ controls (*Gria1*^*Ctrl*^). For the transgenic approach, mixed male-female cohorts (11–12 males and 7–8 females per subgroup) were used, while male-only cohorts were used for the viral approach, as the larger cohorts required for mixed-sex cohorts were not logistically possible due to the time requirements of surgeries and the 5-CSWM-task training (see below). Subgroup sizes were chosen according to our previous data in the respective behavioural tests [39–41]. Surgery was conducted as described previously [40] and in Supplementary Methods. Mice were housed in individually ventilated cages (IVC) containing sawdust, sizzle nest and one cardboard ‘house’ (Datesand, UK) as enrichment, throughout. Temperature (ca. 22 °C), humidity (45–65%) and illumination within a 13 h light/11 h dark cycle (with the light phase starting at 7 a.m.) were tightly maintained within the animal holding room. Where possible, mice were kept in groups of 2–5/cage with ad libitum access to water throughout, and to food prior to and in the interludes between experiments that required appetitively motivated learning. For the latter, mice were kept on food restriction with weights at ~85–95% of their average individual weights under ad libitum food access.

### Behavioural testing

Behavioural experiments started at 2 or 7 months of age in the transgenic and AAV cohorts, respectively, and were conducted during the light phase by an experimenter blind to group identity. The T-maze rewarded alternation and the operant delayed-match-to-place (DMTP) 5-choice (5-CSWM) tests of spatial working memory (SWM), the Y-maze test of spatial novelty preference (SNP), the novel object-recognition test (NOR), the elevated plus-maze (EPM) test of unconditioned anxiety, novelty-induced locomotion, nest-building, the 5-choice serial reaction-time task (5-CSRTT) and related operant rule-shift assays were each conducted as previously described [39, 42, 43] (also detailed in Supplementary Methods). Operant tasks were all conducted in custom-designed 5-choice operant boxes [44, 45].

For reciprocal social interaction, the test mouse was exposed to a younger, adult, same-sex novel stimulus mouse in a familiar open field (dark but transparent Type III cage; Tecniplast, G) and interactions were video-monitored and scored in 2 min intervals for either 12 min (transgenic cohorts) or 16 min (AAV-cohort) [40]. For non-reciprocal interaction, a modified 3-chamber task was utilised, similarly to that used previously to assess sociability in mice with impaired synaptic release from CA2-excitatory cells (see Fig. 4f) [37]: all mice were habituated to the situation of a stimulus mouse by being placed in a half-circular compartment (9 cm radius, perforated metal) at either end of the 3-chamber testing box (50 cm long, 20 cm wide, 25 cm high grey PVC walls) for 2 × 5 min on the previous day and then again once more for 5 min 1–2 h before testing on the first test day. In the first test session, mice were first habituated to the arena without metal compartments (5 min), then to the arena with empty metal compartments (5 min), and were then exposed to a cagemate in one of the empty metal compartments while a mouse-sized piece of black foam was introduced to the other compartment (social preference test, 5 min). The second testing session was conducted on the next day and consisted of four phases: (i) 5 min habituation to the 3-chamber box with empty metal compartments, (ii) 5 min exposure to an unfamiliar stimulus mouse in one compartment and the same cagemate that they had seen on the previous day in the other compartment to assess social novelty preference based on long-term memory (the position of the familiar mouse was counterbalanced within subgroup); (iii) 10 min habituation to the novel mouse from phase 2 (with the other compartment empty) and (iv) 5 min exposure to the mouse from phase 2 and an unfamiliar stimulus mouse to assess social short-term memory (the position of the novel mouse was counterbalanced within groups).

### Statistical analyses

Prior to analysis, some animals were excluded due to insufficient bilateral expression of AAV-transduced Cre (3 dPFC-KO, 2 HC-KO mice), or, in each individual behavioural assay, due to insufficient participation in the assay. Statistical analyses were performed using SPSS (IBM). Two-way repeated-measures analyses of variance (two-way RM-ANOVA) was used in experiments that included multiple challenge protocols, training data, or a baseline measure before a challenge. One-way univariate ANOVA was used on normally distributed data with a simple between-subjects design. Non-parametric Kruskal-Wallis ANOVA or the Mann–Whitney *U* (MWU) test were used, as applicable, when the normality of the data was not assumed. A *P* < 0.05 was considered statistically significant. All data are presented as mean values ± standard error (s.e.m.) or as dot plots representing the values of each individual animal. Detailed statistical results for all experiments are shown in Supplementary Tables 1–4.

## Results

### Spatial working memory impairment in mice with hippocampal ablation of GluA1

**Fig. 1:**
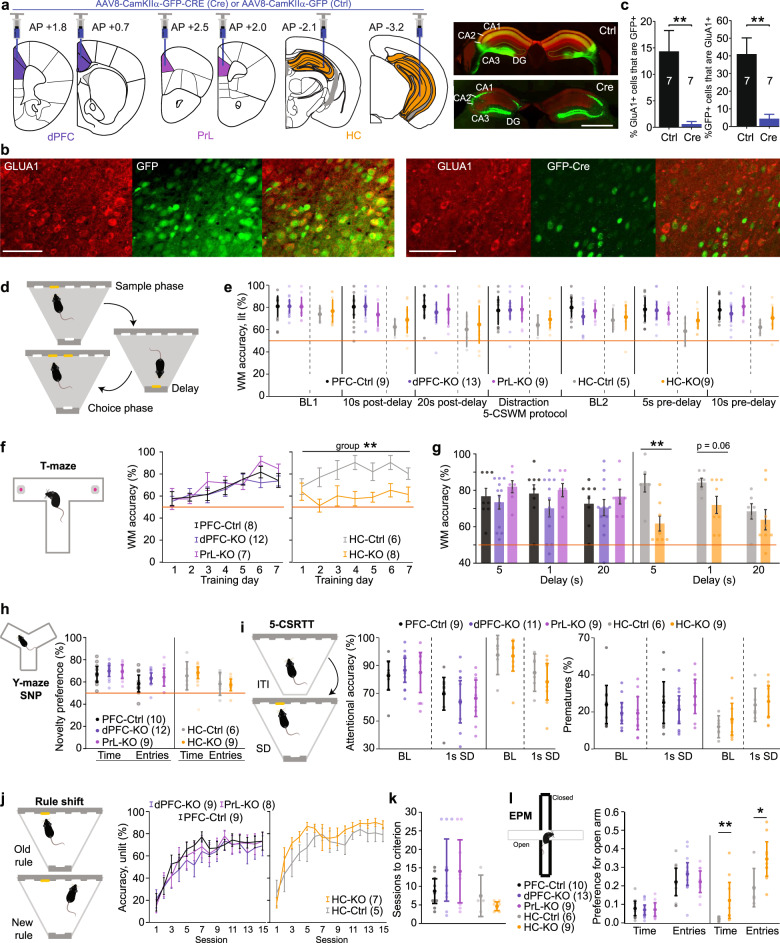
Behavioural phenotyping after rAAV-Cre-mediated ablation of GluA1. **a** Illustration of viral transductions in the regions indicated by colour and named below the coronal section drawings; two bilateral injection sites were used in each group to cover the target structure. Right: Example epifluorescence microscopic images of dorsal hippocampus slices transduced with GFP only (Ctrl, top), or with GFP-Cre (Cre, bottom) and stained against GluA1 (red). See Supplementary Fig. 1 for images from other regions. Scale bar, 1 mm. **b** Confocal images of prefrontal cortex tissue transduced with GFP (left) or GFP-Cre (right) showing GFP (green) and GluA1 (red) expression. Scale bar, 100 μm. **c** Fraction of GFP-positive among GluA1-positive cells (left) and the reverse (right) in prefrontal tissue from the groups identified on *x* axes. **d** Illustration of 5-CSWM task. **e** WM performance across a sequence of 5-CSWM task protocols (named on *x* axis) including two instances of the baseline protocol (BL1/2), and extensions of the delay after or before the reward collection that occurs after the sample phase poke (post- and pre-delay, respectively) by the indicated time. **f**, **g** WM performance during the 7 initial training days in the T-maze rewarded alternation task (**f**) and in protocols with distinct delays (**g**); the data shown for the 5 s delay are the average of the last 3 training days shown in (**f**); the 1 s delay was tested with an ITI of 20–25 s, while the ITI was 5–7 min for the other protocols. **h** Preference for the novel arm in the test phase of the Y-maze test calculated from residence time or the number of entries, as indicated. **i** Two main indicators of attention (accuracy) and impulsivity (premature responding) measured on the baseline protocol and the attention challenge (shortening of stimulus duration, SD, to 1 s) of the 5-CSRTT. Mice with an accuracy <50% at baseline were excluded. **j** Performance according to the new rule (poking of unlit holes in trials where the alternative hole is lit) in a visual rule-shift learning task performed as modification of the 5-CSRTT, for 15 training sessions. **k** Number of sessions needed to reach criterion upon rule-shift learning. **l** Preference for the open arms of the elevated plus-maze calculated from residence time or the number of entries, as indicated. In (**e**–**l**), the five groups are coded by the same respective colour, as indicated in the legend. Groups with prefrontal and hippocampal transductions each had their own control groups and were treated separately for statistics. Significance indicators represent results of RM-ANOVA for time-series data (**f**, **j**), univariate ANOVAs (**e**, **g**, **i**, **k**, **l**) or MWU-test (**c**) for the remainder; see Supplementary Table 1 for exact statistical results. *N*-numbers of mice for each analysis are stated in colour legends or bars in each panel (legend in (**f**) applies to (**g**)). Line and bar graphs represent mean ± s.e.m., all other mean ± C.I., dots show individual subjects. **P < 0.01, *P < 0.05.

To assess if hypofunction of GluA1-containing AMPARs in excitatory cells of the prefrontal cortex or the hippocampus causes schizophrenia-related deficits, we virally transduced either dorsal PFC (anterior cingulate cortex, ACC, and upper prelimbic cortex, PrL), PrL more exclusively, or the hippocampus of *Gria1*^*f/f*^ mice with either GFP-tagged Cre recombinase (Cre groups) or GFP only (control groups) driven by the CamKIIα-promoter, bilaterally (Fig. 1a and Supplementary Fig. 1). For analysis, mice with prefrontal transductions were treated as a separate cohort from mice with hippocampal injections, each with their own control group. Cre-transduction led to a strong reduction of GluA1-positive cells (Fig. 1a–c and Supplementary Fig. 1). As is typical for virus-mediated transductions, expression was mosaic across the cell populations of the targeted areas. With our hippocampal injections, there was minimal bilateral coverage of the CA1 subfield or of the ventral hippocampus, but dorsal CA2/3 was transduced bilaterally in every mouse (Fig. 1a and Supplementary Fig. 1).

After recovery from surgery, rAAV-transduced *Gria1*^*f/f*^ mice were first trained in the DMTP operant working memory task (5-CSWM) which involved multiple stages [42]. In this operant task, mice need to memorise which one out of five holes they had poked into during the prior sample phase while shuttling to the opposite wall of the operant box during the delay, and then poke into the same hole in the choice phase (Fig. 1d). After training, mice were tested with different challenge protocols placing high demands on working memory performance as a result of either distractions during the delay phase or increased delay. Cre-transduced mice of any of the hippocampal or prefrontal groups did not differ from their respective control groups in any protocol (Fig. 1e; see Supplementary Tables 1 and 2 for statistics on this and all subsequent tests in the AAV-cohort).

Later, these mice were trained in the T-maze rewarded alternation SWM task, which utilises a delayed-non-match-to-place paradigm [33]. While Cre-transduction into dPFC and PrL did not affect performance during training or with different delay-challenge protocols in this task, Cre-transduction in the hippocampus impaired performance over the training period (*P* = 0.0036, effect of the group; repeated-measures ANOVA across 7 training days; Fig. 1f, g). While the effect of the group remained a trend with a 1 s delay massed-trials protocol (*P* = 0.058, univariate ANOVA), it was not detected with a longer delay of 20 s reflecting the reduced performance in controls (Fig. 1f, g). In contrast to global *Gria1*^*−/−*^ mice [23, 33], however, performance was significantly above chance level under all conditions (*P* < 0.05, one-sample *t*-test). In the Y-maze spatial novelty-preference test, there was no impairment in the hippocampal group (Fig. 1h).

To test whether sustained attention was affected, mice were trained in the 5-CSRTT, in which mice need to wait and detect a brief illumination of one of the holes in the 5-choice wall in order to be rewarded [39, 43]. This task resembles the sample phase of the 5-CSWM task on which the mice had been trained before. There was no effect of group on measures of sustained attention, task engagement, motivation, perseveration, or impulse control assessed in this task in the baseline training protocol nor in a protocol in which sustained attention was challenged by reduction of the stimulus duration to 1 s applied on the next day (Fig. 1i and Supplementary Table 1). Subsequently, the task was modified to a simpler two-choice task with a longer stimulus duration (8 s). Once mice responded to the illuminated hole with an accuracy of 80% on 2 consecutive days, the task rule was changed to assess cognitive flexibility. Under the new rule, poking of one specific hole of the 5-choice wall (hole 2 or 4) was rewarded irrespective of whether it was illuminated. Cre-transduced mice of all groups learned the new rule at a similar speed as their respective GFP-transduced controls (Fig. 1j, k).

Finally, we assessed several non-cognitive behavioural functions. Both, unconditioned anxiety on the EPM and— in stark contrast to the hyperlocomotion phenotype of *Gria1*^*−/−*^ mice [46]—novelty-induced locomotion in an open field were reduced in mice with hippocampal GluA1 knockout compared to their control group, but were unaffected by prefrontal GluA1 ablation (Fig. 1l and Supplementary Fig. 2). In contrast, reciprocal social interaction was unaltered by hippocampal GluA1 ablation but showed a trend towards an increase with prefrontal GluA1 ablation (Supplementary Fig. 2).

In summary, we found no evidence that GluA1 hypofunction in excitatory cells of the prefrontal cortex contributes to cognitive deficits related to schizophrenia in the battery of tests adopted here, whereas reduction of GluA1 in hippocampal excitatory cells may be responsible for at least some of the short-term memory deficits seen in this disorder.

### *Gria1*-knockout in excitatory cells of CA2 or CA3 causes mild hyperlocomotion

**Fig. 2:**
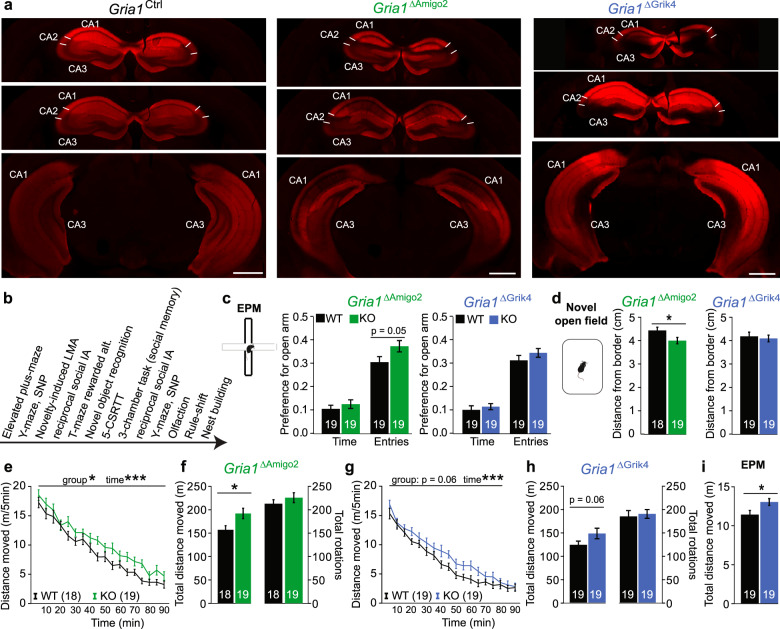
Ablation of GluA1 from CA2/CA3 mildly elevates locomotion. **a** Confocal images of anti-GluA1 staining (red) of slices of dorsal (top) and ventral (bottom) hippocampus from the groups named above. Scale bar, 1 mm. **b** Chronological series of behavioural tests conducted in the transgenic mixed-sex cohorts. **c** Preference for the open arms of the elevated plus-maze (EPM) calculated from residence time or the number of entries, as indicated. **d** Average distance from border during 90 min locomotor activity measurement in a novel open field. **e**–**h** Novelty-induced locomotor activity displayed in 5 min intervals (**e**, **g**) or as total distance moved in 90 min (**f**, **h**). **i** Distance moved on the EPM. In (**c**–**i**), the cohort is identified above the panels and colour-coded, with KO mice and control groups of each cohort in colour and black, respectively. *N*—numbers are stated in colour legends or bars of each panel. The two cohorts were treated separately for statistics. RM-ANOVA was used for analysing data over time (**e**, **g**), univariate ANOVAs for the remainder, ANOVAs used genotype and sex as independent variables; see Supplementary Tables 3 and 4 for exact statistical results. All graphs represent mean ± s.e.m. ***P < 0.001, *P < 0.05.

To further elucidate the potential role of hippocampal GluA1-containing AMPARs, we employed a transgenic approach to achieve more complete targeting of specific populations of excitatory cells, namely those of either the CA2-subfield (targeted by the Amigo2-Cre-driver line [37]; *Gria1*^*ΔAmigo2*^) or of the CA3 subfield (targeted by the Grik4-Cre-driver line [38]; *Gria1*^*ΔGrik4*^). These two hippocampal regions were implicated in spatial short-term memory by our previous rescue study in *Gria1*^−/−^ mice [33], as well as by the experiments described above (Fig. 1f). *Gria1*^*ΔAmigo2*^ and *Gria1*^*ΔGrik4*^ mice showed a noticeable attenuation of GluA1 expression in the CA2 and CA3 subfields, respectively (Fig. 2a).

Given the prior null results of the AAV-cohort in the labour-intensive 5-CSWM task, this task was omitted from the test battery, and unrewarded exploration-based assays were conducted first (Fig. 2b). Like mice with virally mediated GluA1 ablation in the hippocampus (Fig. 1l), *Gria1*^ΔAmigo2^—but not *Gria1*^*ΔGrik4*^—mice showed a marginally higher preference for entries into the open arm of the EPM, potentially indicating reduced anxiety (Fig. 2c; see Supplementary Tables 3 and 4 for statistical assessment of all behavioural tests conducted in the transgenic cohorts). However, they also displayed a reduced average distance from the sidewall of the open field, which could indicate higher anxiety (Fig. 2d). This suggests that there is no clear phenotype for anxiety levels in *Gria1*^*ΔAmigo2*^ mice, reminiscent of findings from global *Gria1*^−*/−*^ mice [46]. Both *Gria1*^*ΔAmigo2*^ and *Gria1*^*ΔGrik4*^ mice displayed a mild increase in novelty-induced locomotion in both the open field and EPM (Fig. 2e–i), although far from the pronounced hyperlocomotion phenotype consistently seen in *Gria1*^−*/*−^ mice exposed to spatial novelty [26, 33, 47].

### GluA1 hypofunction in CA2 and CA3 impairs distinct modalities of short-term memory

**Fig. 3:**
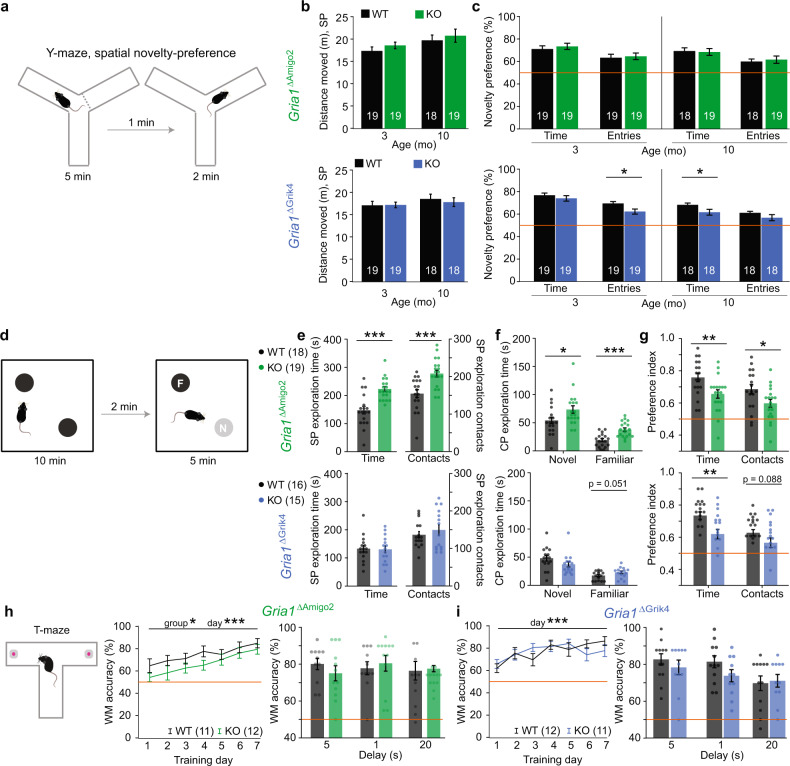
Ablation of GluA1 from CA2/CA3 impairs short-term memory. **a** Illustration of Y-maze SNP task of spatial short-term habituation. **b** Locomotor activity in the sample phase (SP) for the cohorts stated on the left tested at different ages (*x* axes). **c** Preference for the novel arm in the test phase of the Y-maze, conducted at the indicated ages, calculated from residence time or the number of entries, as stated. **d** Illustration of the novel object-recognition test (NOR) of object-related short-term habituation. **e** Exploration of the two identical objects in the sample phase measured as interaction time (left) and the number of contacts (right). **f** Interaction time with novel and familiar object in the NOR choice phase (CP). **g** Preference for the novel object calculated as interaction with novel object relative to interaction with both objects combined using interaction time or the number of contacts as the indicator. **h**–**i** WM performance during the 7 initial training days in the T-maze rewarded alternation task (**h**) and in protocols with distinct delays (**i**); the data shown for the 5 s delay are the average of the last 3 training days shown in (**h**); the 1 s delay was tested with an ITI of 20–25 s, while the ITI was 5–7 min for the other protocols. In (**b**, **c**, **e**–**i**), the cohort is identified above or to the left of the panels and colour-coded, with KO mice and control groups of each cohort in colour and black, respectively. *N*−numbers are stated in colour legends or bars of each panel; the two cohorts were treated separately for statistics. RM-ANOVA was used for analysing data over time (**h**, **i**), univariate ANOVAs for the remainder using genotype and sex as independent variables; the T-maze was only conducted in males. See Supplementary Tables 3 and 4 for exact statistical results. Dot graphs represent individual mice, all other show mean ± s.e.m. Orange line indicates chance level, where applicable. ***P < 0.001, **P < 0.01, *P < 0.05.

Short-term habituation to sensory stimuli is markedly impaired in global *Gria1*^−/−^ mice, resulting in deficits of object-related and spatial novelty preference in the novel object-recognition and Y-maze tests, respectively [7, 21, 22]. We conducted both tests in the *Gria1*^*ΔAmigo2*^ and *Gria1*^*ΔGrik4*^ mice. GluA1 ablation from CA3—but not CA2—excitatory cells caused reduced spatial novelty preference in terms of entries into the novel vs. the familiar arm at a younger age and in terms of time spent in the novel vs. the familiar arm at the older age (Fig. 3a–c). At an older age, spatial novelty preference also differed in dependence on the sex of the mice, which was otherwise not observed in most behavioural tests where females were also assessed (Supplementary Tables 3 and 4 and Supplementary Fig. 3).

In contrast, a deficit in novel object recognition was seen in both *Gria1*^*ΔAmigo2*^ and *Gria1*^*ΔGrik4*^ mice. Notably, *Gria1*^*ΔAmigo2*^ mice also displayed strongly increased exploration of objects in all phases of the task, arguing for a potentially stronger deficit of object-related short-term habituation in these animals (Fig. 3d–g).

Given the profound rewarded alternation deficit in the T-maze in mice with virally mediated GluA1 ablation in the hippocampus (Fig. 1f), we conducted the same T-maze training and testing schedule in the transgenic cohorts. Surprisingly, there was no consistent deficit in either one of these two groups, apart from a mildly reduced performance during the initial T-maze training in *Gria1*^*ΔAmigo2*^ mice (Fig. 3h, i).

### Altered social behaviour and social memory in *Gria1*^ΔAmigo2^ mice

**Fig. 4:**
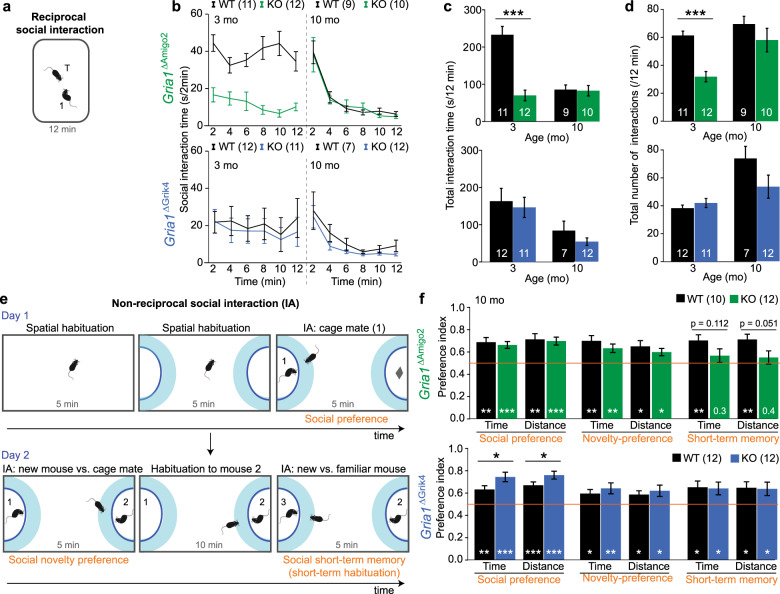
Ablation of GluA1 from CA2/CA3 alters social behaviour. **a** Illustration of reciprocal social interaction assay; see 'Methods'. **b** Social interaction time in 2 min of intervals in the cohorts named on the left for the 12 min of exposure, tested at two different ages (stated in the upper left corner). **c** Same as (**b**) but summed up total interaction time. **d** Total number of interactions in 12 min. **e** Illustration of phases of 3-chamber task (omitting habituation to small compartments on the prior day and a 5 min habituation phase on day 2); see 'Methods'. **f** Preference indexes for the three test phases named in orange in (**e**, **f**) each calculated either from the time spent in the interaction zone around the lateral compartments (cyan in (**e**)), or from the distance the head of the animal moved inside the interaction zone, as stated. The social preference refers to the preference for the compartment with a cagemate compared to the compartment with a mouse-sized object, as determined in the last phase of day 1. Novelty preference refers to the preference for an unknown mouse compared to a cagemate. Short-term memory refers to the preference for another novel mouse compared to the mouse that has become familiar across the two immediately prior phases on day 2. All data shown in (**b**–**d**, **f**) are from male mice, and the cohort is identified on the left of (**b**, **f**) and colour-coded, with KO mice and control groups of each cohort in colour and black, respectively. *N*-numbers are stated in colour legends or bars of each panel, see Supplementary Tables 3 and 4 for statistics; the two cohorts were treated separately for statistical comparison between wild-type and control data with univariate ANOVAs. White stars and numbers indicate the result of the one-sample *t*-test against chance level (orange line). See Supplementary Tables 3 and 4 for exact statistical results. All graphs show mean ± s.e.m. ***P < 0.001, **P < 0.01, *P < 0.05.

Ablation of synaptic release from Amigo2-positive neurons in the hippocampus was shown previously to produce a deficit in social short-term memory [37]. But whether AMPARs in CA2 pyramidal cells are involved in social behaviour is unknown. Therefore, we first conducted a reciprocal sociability test in a familiar environment at 3 months of age. We found that *Gria1*^*ΔAmigo2*^—but not *Gria1*^*ΔGrik4*^—mice showed strongly reduced social interaction (Fig. 4b–d). At 10 months of age, we repeated the test and found sociability to be normal (Fig. 4b–d), possibly suggesting an age-dependent phenotype. At this later age, we also applied the 3-chamber task to assess both sociability and social memory. The task was designed to discriminate between alterations of genuine social short-term memory and alterations of social novelty preference, as such, by testing separately for the preference for a novel mouse over a long-term cagemate [37] (see 'Methods'; Fig. 4f). During the sociability phase, we found that *Gria1*^*ΔAmigo2*^ mice were normal, but *Gria1*^*ΔGrik4*^ mice showed increased social interaction (Fig. 4g). (We have reported previously that global *Gria1*^*−/−*^ mice may show very distinct levels of social interaction, depending on the level of prior habituation to the social arena [19]—a phenomenon, that may underlie the discrepancies between the different social tests conducted in this study.) While both transgenic GluA1-ablation models showed a normal preference for a novel conspecific over a known cagemate, only *Gria1*^ΔAmigo2^ mice showed a deficit in social short-term memory indicated by a chance-level preference score (*P* > 0.3, one-sample *t*-test; note that due to high variability the difference to control mice reached only trend level; Fig. 4g). Their social memory deficit was unlikely to reflect an olfactory impairment, as we did not find an olfactory deficit in *Gria1*^*ΔAmigo2*^ mice (Supplementary Fig. 3). As a related aspect of social behaviour, we also assessed nest-building—but found no alteration in animals of either one of the lines (Supplementary Fig. 3).

### GluA1 ablation from CA3 excitatory cells enhances sustained attention and impulse control

**Fig. 5:**
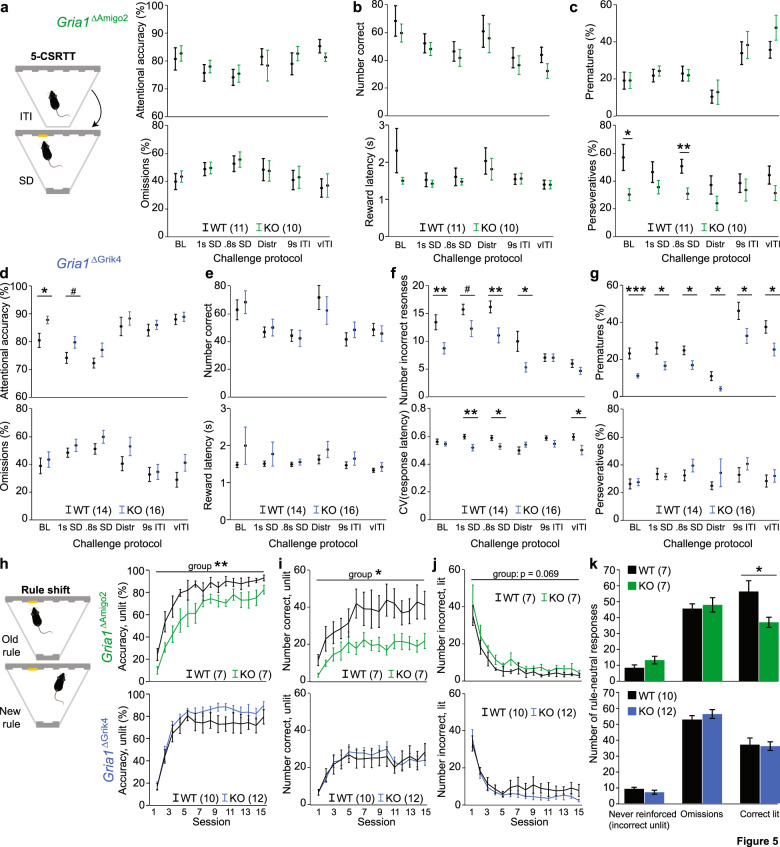
Ablation of GluA1 from CA2/CA3 affects sustained attention, impulse control and cognitive flexibility. **a**–**g** Illustration (**a**, left) and key performance parameter of the 5-CSRTT in *Gria1*^ΔAmigo2^ (**a**–**c**) and *Gria1*^ΔGrik4^ mice (**d**–**g**); including indicators of sustained attention (accuracy) and task engagement (%omissions; **a**, **d**), motivational drive (number correct responses, reward latency; **b**, **e**), impulsivity (%prematures) and perseveration (%perseverative responses; **c**, **g**). Given the increase of attentional accuracy in *Gria1*^ΔGrik4^ mice, additional attentional parameters are shown for this cohort (**f**): the number of incorrect responses and the variability of response latency (coefficient of variation, CV). In all cases, performance is shown for the baseline protocol (BL) and five additional challenge protocols challenging either mainly attention (reduction of stimulus duration, SD, to 1 s or 0.8 s, or auditory distraction, Distr) or mainly impulse control (fixed 9 s or variable extension of the intertrial interval, ITI, waiting time) as indicated on *x* axes. **h**–**j** Key parameters of learning progress in the rule-shift assay in cohorts indicated in (**h**) as extracted from those 50% of trials in each session in which the old and the new rule are in conflict because the correct hole is not illuminated while the incorrect one is; including accuracy of choice for correct non-illuminated hole (the number of correct choices divided by the number of correct and incorrect (lit) choices; **h**), the total number of correct responses into the unlit hole (**i**), and of incorrect pokes into lit hole (**j**). **k** Average number of those responses that are not affected by the rule shift across the first 15 sessions. *N*-numbers are stated in the colour legends of each panel. Cohorts are mixed sex in both cases and animals that have not reached criteria on or before the baseline stages of each task were excluded from testing. The two cohorts were treated separately for statistical comparison between wild-type and control data. RM-ANOVA was used for analysing data over time (**h**–**j**), univariate ANOVAs for the remainder. See Supplementary Tables 3 and 4 for the statistical results. All graphs show mean ± s.e.m. ***P < 0.001, **P < 0.01, *P < 0.05, ^#^*P* < 0.1.

To assess the effects of hippocampal GluA1 ablation on sustained attention, the transgenic cohorts were trained in the 5-CSRTT. Once they achieved stable baseline performance on the final training protocol (stage 5), the mice were tested in five different challenge protocols that demanded elevated sustained attention (due to a reduction of the stimulus duration to 1 s or 0.8 s, or pseudo-random auditory distraction) or impulse control (either due to a fixed extension of the ITI to 9 s or a pseudo-randomly varied extension of the ITI-duration; Supplementary Table 6). *Gria1*^*ΔAmigo2*^ mice did not show any major alterations across the parameters assessed by the 5-CSRTT including parameters for sustained attention (accuracy), impulsivity (premature responding), task engagement (omission rates), motivation (reward latency), or general performance (number correct responses; Fig. 5a–c). The exception to this was a reduction of perseverative responding seen in two protocols (Fig. 5c and Supplementary Table 3).

In striking contrast, *Gria1*^ΔGrik4^ mice showed a consistent reduction of premature responding across protocols and a reduction in the number of incorrect responses in most attention-related challenges and in the baseline protocol, driving an improvement of attentional accuracy (Fig. 5d, e and Supplementary Table 4). Even the variability of response latencies—which is the primary attention parameter in the human version of this task (although not commonly used in rodents)—was reduced by GluA1 ablation in CA3 in two of the attention challenges and the vITI condition (Fig. 5f). Reward latency, the number of correct responses, omissions and perseveration were not significantly altered. In summary, this overall pattern of a selective reduction of those active response types that are erroneous suggests an improvement in aspects of impulse control and sustained attention in *Gria1*^*ΔGrik4*^ mice.

### Decreased cognitive flexibility in *Gria1*^ΔAmigo2^ mice

A related psychological function is cognitive flexibility, one form of which is the capacity to recognise and learn a changing rule. To assess visual rule-shift learning, we trained the mice on a simplified form of the 5-CSRTT, applying an extended stimulus duration and involving only two choices. We then switched the rule from one where the reward was dependent on the light cue to one based on a spatial association with the reward (with the hole illumination now being irrelevant, as described previously for the AAV-cohort; Fig. 1j) [39]. In contrast to the results from the 5-CSRTT, *Gria1*^*ΔGrik4*^ mice—although scoring qualitatively higher than their respective control group—were not significantly better at rule-shift learning by any metric (Fig. 5h–k and Supplementary Fig. 3). However, *Gria1*^*ΔAmigo2*^ mice performed significantly worse than their controls across the first 15 d of testing with the new rule (Fig. 5h). While their perseverative responding (errors that would have been correct responses by the old rule) was marginally higher, the main driver for their reduced accuracy was a strong reduction in correct responses in trials that required the application of the new rule (Fig. 5i–j). This implies that mice were able to recognise that the rule had changed but were less capable of acquiring the new rule—a phenomenon we have observed before in mice with elevated hippocampal activity [39]. Even the number of correct responses on trials where old and new rules were not contradicting each other (i.e., the spatially correct hole was also lit) was decreased, indicating reduced ability to grasp the overall rule-set.

## Discussion

In this study, using different ablation models, we show that GluA1-containing AMPARs in hippocampal—but not prefrontal—excitatory cells are relevant for a wide range of cognitive functions across a behavioural spectrum relevant to schizophrenia (summarised in Table 1). Most prominently, GluA1 ablation in excitatory cells of the hippocampus impaired short-term memory, whereby the specifically affected modality—spatial, object-related or social memory—depended on the manipulated hippocampal subfield. Strikingly, hippocampal GluA1 ablation could entail not only impairments but also enhancements of behavioural performance (Table 1). These results have multiple neuropsychological and translational implications.

**Table 1:**
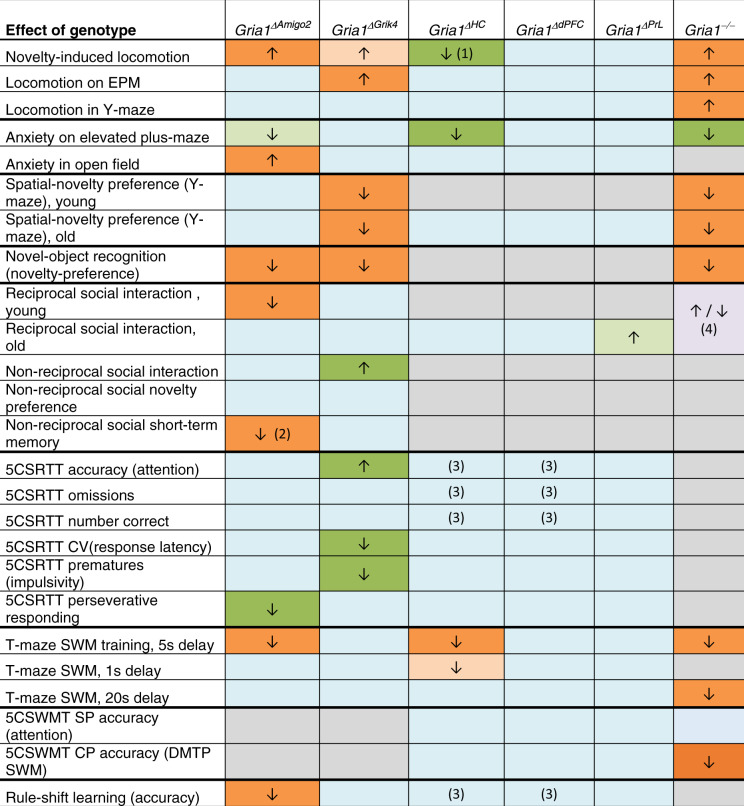
Summary of behavioural results in transgenic and AAV cohorts.

*CV(response latency)* coefficient of variation of response latencies, *CP* choice phase, *SP* sample phase, *SWM* spatial working memory.

The difference between the respective *Gria1*-ablation mouse model identified in the first row and their respective control groups (Cre-negative littermate fGluA1 mice for *Gria1*^Δ*Amigo2*^ and *Gria1*^Δ*Grik4*^, littermate wild-type mice for *Gria1*^−/−^ and GFP-transduced littermate fGluA1 mice for the remainder; see 'Methods') are represented for the behavioural tests and variables stated in the leftmost column. Published data from the global *Gria1*^*−/−*^ is shown for comparison in the right column [19, 21, 23, 24, 33, 46, 50]. ↑ increase of behavioural parameter; ↓ decrease of behavioural parameter. Colour-code: grey, not tested; blue, no significant effect of genotype; orange, deficit; light orange, the trend for a deficit; green, improvement; light green, the trend for an improvement. Comments: (1) decrease only relative to hippocampal controls (not relative to the prefrontal control group). (2) Difference to control group only reaches trend level, but only KO mice show the absence of social novelty preference. (3) *Gria1*^Δ*HC*^ mice perform significantly better than *Gria1*^Δ*dPFC*^ mice. (4) Behavioural changes depend on prior habituation to the arena [19].

### Causal association of short-term memory with GluA1 in distinct hippocampal subfields

Despite some degree of variability across experiments, an otherwise clear picture emerges of impaired short-term memory in mice lacking GluA1 in the CA2 and/or CA3 hippocampal subfields (Table 1). This is consistent with our previous study demonstrating a rescue of a form of spatial short-term memory, assessed by spatial novelty preference, with the reintroduction of GluA1 into this region [33]. Notably, in a parallel study using transgenic mice in which GluA1 was ablated from both CA1 pyramidal cells and dentate gyrus granule cells under the control of the CN-12 promotor line [48], we have found no evidence of any short-term memory impairment (e.g., on the T-maze rewarded alternation task or the Y-maze spatial novelty-preference task; Lee et al., in preparation). This suggests that GluA1-dependent synaptic plasticity in the CA2/CA3 subfields may be particularly important for these short-term memory processes (as discussed previously [33]). That said, it is also important to point out that the magnitude of the memory deficits reported here (and before [36]) in regionally selective knockouts are considerably less than what we have reported previously in global GluA1-knockout animals [21–24]. This suggests a combinatorial effect whereby GluA1 elsewhere in the brain is also relevant for short-term memory which may allow for compensation following the loss of GluA1 in one specific hippocampal subfield.

Nevertheless, the variable patterns of impairments and sparings in the different hippocampal knockout models we have used, across various tasks, are somewhat surprising and not easily explained. For example, within assays of spatial short-term memory, GluA1 hypofunction specifically in the CA3 pyramidal cells of *Gria1*^*ΔGrik4*^ mice reduced spatial novelty preference in the Y-maze but not T-maze rewarded alternation whereas more widespread, but less complete, GluA1 ablation across principal cells of all hippocampal subfields in the *Gria1*^*ΔHC*^ mice impaired T-maze SWM performance but not Y-maze novelty preference. These tasks may differ in their sensitivity, the cues that are used, as well as the psychological processes that may be required. Importantly, the clear dissociation between the T-maze rewarded alternation task and the spatial novelty-preference Y-maze task suggests that, at least under some circumstances, rewarded alternation may not merely reflect the spatial short-term habituation processes that support spatial novelty preference [49], and, conversely, the latter does not necessarily reflect spatial working memory. Likewise, virally mediated hippocampal GluA1 hypofunction did not worsen spatial operant DMTP working memory, but alternation-based DNMTP working memory on the T-maze— which is the reverse phenotype of what we have observed with prefrontal NMDAR-ablation [40] and which confirms that these two spatial working memory tasks are mediated by different mechanisms, as we recently suggested based on physiological data [50].

We have argued previously that impaired short-term habituation may be a key pathological mechanism towards the aberrant assignment of salience in schizophrenia [7, 20, 25, 27, 51] and that GluA1 in CA2/CA3 is key in regulating spatial short-term habituation via functional connectivity in the theta frequency range (theta coherence) [33]. The present data suggest that it is GluA1 in CA3, rather than CA2, that supports spatial short-term habituation (as indicated by reduced Y-maze spatial novelty preference in the *Gria1*^*ΔGrik4*^ mice), although even the *Gria1*^*ΔGrik4*^ animals were markedly less affected than global *Gria1*^−*/−*^ mice [24, 33, 47]. In contrast, short-term memory for *objects* was affected more severely in *Gria1*^*ΔAmigo2*^ mice compared to *Gria1*^*ΔGrik4*^ mice (as evident from their increased object exploration, not seen in the latter group), and short-term memory for social stimuli was impaired *only* in *Gria1*^*ΔAmigo2*^ mice. These results suggest that the short-term memory mechanisms underpinning short-term habituation in the hippocampus may be supported by different hippocampal circuits depending on the type of stimulus that is being processed, which presumably reflects differences in the information that is received by the CA2 (social novelty) and CA3 (contextual novelty) subfields and their distinct anatomical inputs [52, 53]. Altogether, our results demonstrate that seemingly similar psychological functions are dissociable to a surprising degree.

### Possible cognitive functions of GluA1 in CA2-excitatory cells

Excitatory cells of CA2 have been implicated previously as central to social short-term memory [37, 54, 55]. Our present data confirm this association, demonstrating impaired social short-term memory in *Gria1*^*ΔAmigo2*^ mice (Fig. 4g), and suggest that this cognitive function relies on GluA1-dependent synaptic plasticity within CA2-excitatory cells. Interestingly, our results also point *beyond* a social function of CA2. In line with a more general role of CA2 in novelty-processing [55], *Gria1*^*ΔAmigo2*^ mice also displayed mildly elevated spatial novelty-induced hyperlocomotion, strong increases of object exploration and impairments of novel object recognition (i.e., novel object preference), mild deficits during the acquisition of the T-maze rewarded alternation task and the rule-shift assay. This phenotypic pattern points to a principle difficulty in the appropriate processing of salient stimuli, a central pathology in schizophrenia (often referred to as aberrant salience [25, 27, 51]) which can lead to maladaptive learning, extending beyond the social domain. Notably, impaired performance in short-term memory tests and in reversal learning (a different measure of cognitive flexibility) correlates with impaired salience attribution in patients with schizophrenia [56–58] suggesting a common, possibly salience-related mechanism.

Given that Amigo2 expression is more extensive both inside and outside the hippocampus early during development [37], with our use of a transgenic approach (to ensure complete GluA1 ablation in CA2) we cannot completely rule out the possibility that GluA1 ablation outside CA2 (although, evidently not in the prefrontal cortex, Fig. 1) contributed to this phenotype. The extent of any non-hippocampal ablation is difficult to evaluate due to the much lower native GluA1 expression outside the hippocampus (Fig. 2a), the mosaic and time-dependent distribution of Amigo2 expression during development [37], and the dependence of the stochastic Cre-lox-recombination on the distance between lox-sites [59] which is smaller in typical fluorescent reporter lines (e.g., 838 bp in the widely used Ai9 and Ai14 tdTomato Cre-reporter lines [60]) than in the floxed *Gria1* line (1628 bp) [35]. Therefore, the possibility of an association between CA2 and processing of non-social stimuli needs to be further scrutinised in future using virally mediated local manipulations [61] in combination with the tasks described here.

### Regulation of impulse control and attention by GluA1 in CA3 excitatory cells

Psychological functions that are necessary for efficient, adaptive goal-directed behaviour include impulse control, sustained attention, and cognitive flexibility. These functions have mainly been associated with prefrontal circuits [62–64], although some evidence for mildly reduced sustained attention and impulse control (as assessed by the 5-CSRTT) resulting from hippocampal manipulations does exist [25, 44, 65–67]. Our finding that *Gria1*^*ΔGrik4*^ mice showed a selective and profound reduction of response time variability and of active erroneous responses that would result from decreased attention or increased impulsivity in the 5-CSRTT pinpoints the CA3 subfield, its excitatory cells and GluA1-AMPARs within these cells as key regulators of sustained attention and impulse control. Since GluA1-AMPARs mediate the early phase of hippocampal long-term potentiation (LTP) [1, 2, 4, 68, 69]—including plasticity at synapses of recurrent excitatory connections within CA3 [70]—this suggests that GluA1-induced short-term synaptic enhancement might be a mechanism for adjusting levels of attention and impulsivity during goal-directed behaviours. Notably, virally mediated ablation of NMDA receptors from excitatory cells in CA3 produced a somewhat reversed phenotype compared to that of *Gria1*^*ΔGrik4*^ mice, with elevated motor impulsivity and reduced social interaction [67].

### Implications for treatment strategies

Surprisingly, our data would seem to suggest that GluA1 hypofunction in excitatory cells is not unequivocally detrimental for cognitive performance. Aside from recurring deficits in short-term memory resulting from hippocampal GluA1-KO, we also observed phenotypes that appear to be the opposite of the reduced sociability and impaired attention observed in patients with schizophrenia [71, 72]. Notably, these phenotypes may reflect the more salient presentation specifically of familiar sensory stimuli as is caused by a failure of short-term habituation in global *Gria1*^*−/−*^ mice and associated with increased dopamine release [7, 20, 22]. In either case, this has important implications for the pharmacological strategy of enhancing AMPAR function in schizophrenia. So far, non-specific pharmacological augmentations of AMPARs have been developed in the form of ampakines, which enhance hippocampal long-term potentiation [73]. In line with our present data, the ampakines *CX516* and *faramptor* can improve short-term memory performance in healthy humans [29, 74, 75], and a more recent clinical trial (NCT01749098) with the ampakine *PF-04958242* demonstrated its ability to improve ketamine-induced working memory deficits in healthy volunteers [76]. However, outside the domain of short-term memory, evidence is more equivocal. For example, the effects of ampakines on long-term memory are mixed [28, 29]. Also, after a promising small clinical trial [30], CX516 was not found to be effective in improving cognition in patients with schizophrenia [30]. Therefore, further development of the ampakine drug class might instead resort to compounds with modulatory effects, rather than agonists, to limit AMPAR enhancement to activity that is endogenously ongoing. For example, the positive allosteric modulator *TAK-137* improved psychostimulant-induced hyperlocomotion, as well as social interaction, working memory, sustained attention, and reversal learning in rodents and non-human primates in the naive state or in pharmacological NMDAR-hypofunction models (MK-801, ketamine) [77]. In line with our present findings, this demonstrates that the restoration of appropriate levels of AMPAR function—and hence GluA1-dependent plasticity—rather than their blunt enhancement, could be therapeutically more effective. Furthermore, the mixed and partly mild behavioural phenotypes in our cell-type-selective *Gria1*-ablation models align not only with such treatment studies but also with genetic studies [18, 78, 79], suggesting that many different molecular alterations can contribute to schizophrenia by causing synaptic dysfunction in the hippocampus. Pharmacological manipulation of one receptor may not be effective in correcting such diverse synaptic pathologies in every patient population.

## Supplementary information

Supplementary Information
