# Supplementary material for: Distinct contributions of GluA1-containing AMPA receptors of different hippocampal subfields to salience processing, memory and impulse control
Source: Transl Psychiatry. 2022 Mar 14;12:102. doi: 10.1038/s41398-022-01863-8 (PMC8921206; doi:10.1038/s41398-022-01863-8)
Supplement: Supplementary file 1 — Supplementary Information [file 41398_2022_1863_MOESM1_ESM.docx]

# Supplementary Information

Distinct contributions of GluA1-containing AMPA receptors of different hippocampal subfields to salience processing, memory and cognitive control

***Running title:* Hippocampal GluA1 AMPA receptors in cognition**

**Keywords:** AMPA receptor, working memory, short-term memory, hippocampus, prefrontal cortex, schizophrenia, mouse

**Authors:**

Kasyoka Kilonzo^1^, Daniel Strahnen^1^, Vivien Prex^1^, John Gems^1^, Bastiaan van der Veen^1^, Sampath K.T. Kapanaiah^1^, Bhargavi K.B. Murthy^1^, Stefanie Schulz^1^, Rolf Sprengel^2,3^, David Bannerman^4^, Dennis Kätzel^1,*^

^1^ Institute of Applied Physiology, Ulm University, Ulm, Germany

^2^ Max-Planck-Institute for Medical Research, Heidelberg, Germany

^3^Institute for Anatomy and Cell Biology, Heidelberg University, Germany

^4^ Department of Experimental Psychology, University of Oxford, Oxford, UK

* Correspondence: dennis.kaetzel@uni-ulm.de; +49 731 500 33770; Fax +49 731 500 33779; Institute of Applied Physiology, Ulm University, Albert-Einstein-Allee 11, 89081 Ulm, Germany

# Supplementary Methods

## Surgery

To generate the AAV-cohorts, stereotactic surgery was performed on fGria1 mice before the behavioural test battery commenced. Animals were anaesthetized using 5% isoflurane (AbbVie, G), received s.c. injections of analgesics (0.08 mg/kg buprenorphine, Bayer, G; 1 mg/kg meloxicam, Boehringer Ingelheim, G), and local scalp anaesthesia (200 μl of 0.025 % bupivacaine, AstraZeneca, UK) before placement in a stereotaxic frame (motorized and atlas-integrated frame, Neurostar, G and Kopf, US; manual digital frame, World Precision Instruments, US) with non-rupture mouse ear bars. The body temperature was stabilized using a feedback-controlled heating blanket (Harvard Apparatus, US) and the anaesthesia was maintained with 1.5 % isoflurane. The following stereotaxic coordinates (from bregma) and volumes were used for bilateral transfection of the stated areas; *dPFC* (targeting anterior cingulate cortex): posterior injection at AP +0.5, ML 0.3, DV 1.7 (200 nl) and 1.2 (300 nl), anterior injection at AP 1.7, ML 0.3, DV 1.25 (80 nl). *PrL*: posterior injection at AP +2.0, ML 0.3, DV 2.1 (50 nl) and anterior injection at AP 2.5, ML 0.3, DV 1.9 (60 nl). *Hippocampus*: anterior (dorsal) injection at AP -2.1, ML 2.3, DV 1.85 from pia (400 nl); posterior (ventral) injection at AP -3.2, ML 2.4, DV 3.6 (300 nl) and 3.3 (100 nl) from pia. All viral vectors were of serotype AAV8, had a titre of 4 x 10^12^ vg/ml, and were obtained from the University of North Carolina vector core (UNC, NC, US). An AAV8-CamKIIα-*GFP-Cre*-WPRE-hGHpA vector was used for *Gria1*-ablation the experimental groups, and a corresponding AAV8-CamKIIα-*GFP*-WPRE-hGHpA vector for the control groups. A pouch to absorb the virus was created by moving the needle 0.05 mm further down and then up again to the actual DV position before infusion. Infusions were made using a glass 10 μl precision syringe (WPI) at an injection rate of 100 nl/min. Upon completion of infusion the needle was kept in place for 5 min, moved up 0.1 mm and kept in place for another 5 min in order to minimize backflow of the virus. Mice received post-operative monitoring for 7 d, and an s.c. injection of the analgesic meloxicam (Metacam, 1 mg/kg, Boehringer Ingelheim, G) on the first 3 d. The mice were kept on *ad libitum* food for a minimum of two weeks before training in the 5-CSWM task commenced.

## DMTP 5-choice spatial working memory (5-CSWM) task

All rewarded tasks (5-CSWM, 5-CSRTT, T-maze) were conducted under food restriction during which subjects were maintained at 85%-90% (5-CSRTT learning in transgenic cohorts) or 90-95 % (all other) of their free-feeding weight. The 5-CSWM was conducted and trained as described previously, albeit in custom-made pyControl-based operant boxes^1,2^ (described in detail on https://github.com/KaetzelLab/Operant-Box-Design-Files). Task scripts for all applied training stages and challenge protocols, including task illustration and description are available on https://github.com/KaetzelLab/Operant-Box-Code. Briefly, all experimental stages of the 5-CSWM and prior habituation training were conducted in the dark, by default. Transient illumination resulted either from the poke holes in the 5-choice wall during cue-presentation, from the receptacle light during reward delivery, or from the house light of the chamber during 5 s time-out (“punishment”) periods after erroneous responses or omissions.

Before the beginning of the training and coinciding with the beginning of the food restriction, mice were accustomed to the strawberry milk reward (Müllermilch, G) in their home cages, and subsequently, in the operant boxes. Initially, mice conducted a 5-light operant training protocol to learn to poke into any illuminated hole to obtain a 40 μl milk reward. Once mice obtained >29 rewards in two consecutive 30 min sessions or a maximum of 20 sessions, training in the actual 5-CSWM task started.

The basic 5-CSWM task operant cycle began with a *sample phase* (SP) during which the mouse had to poke the one hole of the 5-choice wall that was illuminated for a certain *stimulus duration* (SP-SD). If correctly poked within the SP-SD time, illumination of this hole was turned off, the receptacle light was turned, and a 20 μl reward was delivered. A time of 2 s was allowed for consumption and ended by turning off the receptacle light. Immediately afterwards, a *delay phase* of 2 s started and was followed by a *choice phase* (CP) during which - for a maximum *stimulus duration* (CP-SD) of 20 s - the mouse was presented with two illuminated holes at the 5-choice wall, of which one was the hole that had been presented in the prior SP and was the correct choice option in this DMTP paradigm. In case of a correct poke into this hole (*correct response*), the cue lights were turned off and the receptacle was illuminated with simultaneous delivery of a 60 μl reward. All other responses were not rewarded but were followed by a time-out. These erroneous responses included pokes into non-illuminated holes during the SP (*incorrect SP response*) or CP (*incorrect_unlit_ CP response*), incorrect responses into the illuminated hole during the CP (*incorrect_lit_ CP response*), or omissions of any response during the allowed SD response times in either phase (*SP omission, CP omission*). Premature pokes had no consequence. An inter-trial interval (ITI) of, mostly, 5 s (in addition to the 5 s time-out after erroneous responses) followed before a new SP with cue-presentation was started. The ITI was extended to 15 s for 6 sessions *before* and for the sessions *of* the first three challenges.

The primary readouts during this operant cycle were the key measure of WM performance, *accuracy_lit_* (number of correct CP responses divided by the number of correct and incorrect_lit_ CP responses), the CP omission rate (%) as a measure of task engagement (number of CP omissions divided by number of correct SP responses), and – as a metric for stimulus-directed attention, the SP *accuracy* (number of correct SP responses divided by the number of all SP responses, excluding SP omissions). To aid task acquisition, the 5-CSWM was trained in increasingly difficult stages through which the mice transitioned depending on achieving certain performance criteria during 3 consecutive days on the prior stage. The parameters and performance criteria of these stages and later challenge protocols are stated in Supplementary Table 5. Challenges of working memory were implemented by extending the delay phase either *after* the SP-reward collection (*post-delay*) from 2 s to 12 s or 22 s, or *before* the SP-reward delivery (pre-delay) from 2 s (special baseline protocol, baseline 2) to 7 s or 12 s, or by introducing a visual distraction (switching on the house light two times at random during a 5 s post-delay).

## 5-choice serial reaction time task (5-CSRTT)

Mice of the AAV-cohorts that had learnt the 5-CSWM task were transferred to the 5-CSRTT, immediately after completion of the challenge battery. The 5-CSRTT essentially corresponds to the SP of the 5-CSWM task, except that the light conditions are inverted (box illuminated by default and dark during time-outs) and that premature responses during the ITI are punished by a time-out. These mice were directly trained on stage 4 of the 5-CSRTT training stages, whose parameters are stated in Supplementary Table 6. Mice of the transgenic cohorts, in contrast, were trained in the 5-CSRTT starting with the standard operant habituation training (see 5-CSWM section above) and were then trained up through all 5 training stages. The 5-CSRTT was conducted like previously described^3^, except that the same custom-made operant boxes were used that were also used for the 5-CSWM. Task scripts of all training and challenge stages are available on https://github.com/KaetzelLab/Operant-Box-Code. The basic operant cycle of the 5-CSRTT starts with a waiting time (the inter-trial-interval, ITI) of 2 s or 5 s, depending on the training stage, which is followed by illumination of one of the five holes for a certain stimulus duration (SD). If mice poke into that hole during the SD or the subsequent 1 s, the reward receptacle is illuminated and a reward of 20 μl delivered, whose consumption is followed by another ITI. If mice poke before SD onset (premature response), during the SD into a non-illuminated hole (incorrect response), or refrain from poking (omission) reward is withheld and instead a 5 s timeout period is initiated after which a new ITI starts. If mice poke into a correct hole again before collecting the reward, this poke is counted a perseverative response. Key readouts of the task^3,4^ are the primary measures of sustained attention, accuracy (number of correct responses divided by the sum of correct and incorrect responses), of task-engagement (% of trials with omissions), motor impulsivity (% of trials with premature responses), and perseveration (ratio of perseverative responses and correct responses expressed as %). Additionally, reward latency – the time between correct poke and entry into the reward receptacle – is measured as an indicator of locomotor drive, and response latency – the time between SD-onset and correct poke – is measured as an indicator or processing speed. Additionally, in alignment with human versions of the 5-CSRTT, we recorded the variability of the correct response latency as an additional measure of attentiveness (coefficient of variation, CV, equalling the ratio of standard deviation and mean of the individual response latency values of all correct responses).

## Visual rule-shift learning

Rule-shift learning was conducted based on the prior training of mice in the 5-CSRTT, as previously described^3^. Mice were moved to a simplified, 2-choice version of the 5-CSRTT (see above) with an SD of 8 s, an ITI of 2 s, only holes 2 and 4 provided as choice options, and premature responses not punished. Mice were trained in this protocol until they have achieved an accuracy of 70% (AAV-cohorts) or 75% (transgenic cohorts) in at least two consecutive sessions (with a majority of mice scoring >80% before the rule-shift). Subsequently, the rule was changed and a fixed hole (2 or 4, assignment counterbalanced within group) was rewarded, irrespective of illumination. Therefore, in 50% of trials the old and the new rules were not in conflict (rewarded hole illuminated), while in the other 50% of trials the rules were in conflict with each other (rewarded hole was unlit, while the unrewarded hole was lit). A choice accuracy was calculated for those 50% of trials in which the rules were in conflict by dividing the number of correct(unlit) choices by the sum of correct(unlit) and incorrect(lit) choices. Mice were considered to have achieved the criterion of rule-shift learning if their accuracy_unlit_ exceeded 70% on two consecutive days. Irrespective of achieving that criterion, all mice were trained for 15 days with one 45 min session per day.

## T-maze rewarded alternation

The T-maze delayed rewarded alternation task was used as a non-matching to position (DNMTP) paradigm of spatial WM and conducted as previously described^3^. The T-shaped maze (W 10 cm, L 40 cm; H 10 cm) consisted of a red PVC floor and transparent Perspex walls with metal food wells at the end of each goal arm. The reward used for this experiment was a 2:1 dilution of condensed milk (‘Ja’, REWE, G) in their drinking water. First, mice were habituated to the maze in groups (with cagemates) and then individually. A trial of the actual task, started with the sample phase (SP), during which mice were placed in the start arm and allowed to enter a pseudo-randomly determined goal arm to consume the reward, while the alternate arm was blocked. After gaining the reward in the SP, the mouse was removed from the maze for a 5 s delay phase, the barrier blocking the previously inaccessible (novel) goal arm was removed, and then the mouse was returned to the start arm for the choice phase (CP) and left to choose between the novel, rewarded and the familiar, unrewarded arm. Throughout, mice were trained and tested in one session of 10 trials per day. (As an exception to this, mice of the *Gria1^ΔAmigo2^* cohort received two sessions per day starting from the 7^th^ session.) Initially, 7 sessions were applied with a delay of 5 s and an ITI of ca. 5-7 min. Subsequently, mice performed two sessions with a shortened delay of 1 s and an ITI of 20-25 s, and then a further two sessions with an extended delay of 20 s and an ITI of ca. 5-7 min. Some mice were also tested with a 60 s delay subsequently (data not shown). The number of correct choices divided by the number of trials per session was calculated as a readout of WM performance (WM accuracy), and for analysis of different delays the accuracy was averaged across the days with equal delay (see Fig. 1f,g and 3h,i).

## Y-maze spatial novelty preference test

Spatial short-term habituation (a form of spatial short-term memory) was assessed as spatial novelty-preference in a Y-shaped maze with three transparent arms (20 cm high, 30 cm long, 8 cm wide) uniformly separated by 120° as previously described^3^. The floor of the maze was covered with clean sawdust that was interspersed with some dirty sawdust collected from the home-cage of an unfamiliar same-sex group of mice. Before the test, each mouse was kept in a holding cage near the Y-maze for 8-10 min. The test began with a 5 min sample phase (SP) where the mouse could explore the start arm and one goal arm (counterbalanced within groups). The alternate goal arm was blocked by a non-transparent grey PVC barrier. At the end of the SP, the mouse was transferred back into the holding cage for a 1 min delay during which the barrier to the previously inaccessible arm was removed and the saw dust from all arms was mixed and dispersed within the maze. The mouse was subsequently returned to the maze for a 2 min test phase which allowed access to all arms. ANY-maze (San Diego Instruments, US) was used to track the movement of the mouse during the experiment. Novelty preference was analysed by calculating the *time* spent in the novel goal arm divided by the sum of the time spent in both goal arms. Additionally, a preference score was calculated based on the *number of entries* into the novel goal arm divided by the sum of the number of entries into both goal arms.

## Novelty-induced locomotor activity measurement (LMA)

Locomotor activity was tested in a novel open field (OF), which was a clear plastic cage (425 x 266 x 185 mm; Eurostandard Typ III, Tecniplast, G) filled with clean sawdust. Movements were recorded for 90 min using CCTV cameras (Sentient, UK) installed centrally above the open-field cages. The video-recordings were fed into a single image frame through a CCTV-system (Dahua Inc, China), digitized through an A/D converter (TheImagingSource, G), and processed by ANY-maze (San Diego Instruments, US) to extract the distance moved in 5 min time intervals as well as the average distance to the OF border.

## Novel object recognition (NOR)

The NOR test of object-related novelty preference was used to assess short-term habituation (memory) for objects as previously described^3^. Briefly, mice were habituated to the square open-field (dark-red floor, grey PVC walls of 25 cm height, 40 cm length and width) over the two days before testing; the first 5 min habituation session was conducted with the cage mates, all subsequent ones – one time 5 min on the same day, one time 5 min and one time 10 min on the next day, and one time 10 min on the morning of the test day – were conducted with each mouse individually. On the test day, mice were habituated to a holding cage for ca. 20 min and then introduced to the familiar open field in which two copies of the same unfamiliar object were placed (sample phase). After 10 min, the subject was removed into the holding cage for 2 min so that both objects could be replaced, whereby one was replaced by an identical object and the other one by a novel object. Subsequently, mice were introduced to the open field again for the 5 min test phase. Mice were video-monitored and thereby their interaction with objects scored manually, in addition to tracking with ANY-maze. Data from manual scores was used for further analysis. Novelty preference was analysed by calculating the *time* spent in with the novel object divided by the sum of the time spent with either object. Additionally, the same preference score was calculated based on the *number of contacts*.

## 3-chamber test of social interaction and social memory testing

See Methods section of the main text.

## Reciprocal social interaction

All tests used adult, but younger mice of the same sex and strain as stimulus mice. For reciprocal social interaction, the test mouse was exposed to the novel stimulus mouse in a familiar open-field (dark but transparent Type III cage; Tecniplast, G). For the first test in all cohorts, mice were habituated for 90 min to the social arena on the day before testing (this habituation was the novelty-induced locomotor activity test described above). For the second test session in the transgenic cohorts, mice were habituated to the arena for 10 min on the prior day and for 5 min immediately before testing. Interactions were video-monitored and scored in 2 min intervals for either 12 min (transgenic cohorts) or 16 min (AAV-cohort). A shorter time was used in the larger transgenic cohorts to allow their testing within one day per strain and sex, given that no additional meaningful group-difference had emerged in the last 4 min of testing in prior experiments in the AAV-cohort (see Supplementary Fig. 2c) or other studies of ours^3,5^. In the transgenic cohorts, a second 12 min interaction test was conducted at an older age. In this case the test was followed by two consecutive 4 min phases 1 h later during which mice were exposed either to the same or a new stimulus mouse (order counterbalanced within subgroup) to assess social memory^6^ – however, no indication of social memory was seen even in controls in this test, therefore the data is omitted here.

## Olfactory function test

Olfactory function was assessed after 20 min of habituation to an open-field by exposing mice to an odour (rose or lavender) 4 times for 2 min every 4 min whereby on the first three exposures the odour was the same and on the last exposure a novel odour was presented.

## Elevated plus-maze (anxiety)

The cross-shaped elevated plus maze (EPM) was used to test for unconditioned anxiety and was custom-made from grey PVC. Its four arms had identical area (L 35.5 cm, W 7 cm), were connected by a neutral centre (L 7 cm, W 7 cm), and two opposing arms were open, while the other two were enclosed by walls of 20 cm height. The maze was elevated 70 cm above the ground and the area underneath the maze was covered with cloth to protect the mice in the eventuality of a fall during the test. Light intensity in the maze was 100 lux on open arms and 75 lux in the centre. Subjects were at first placed in a novel holding cage in the testing room for a 5-7 min habituation period. Thereafter, the mouse was transferred to the centre of the maze and allowed to explore the maze for 5 min. The movement was tracked and analyzed using ANY-maze. Entries into each zone were defined according to the position of the mouse’s body centre. A preference score was calculated as the time spent in the open arms divided by the time spent in the closed arms (excluding the centre). This measure represents the primary, inverse measure of unconditioned anxiety in relation to exploratory drive.

## Nest building

For nest building assessment, mice were single-housed in cages with just saw-dust covering the floor and a single 2.6-2.7 g Nestlet (Datesand, UK), at the evening (6-7 pm). On the next morning (8-9 am), the resulting nests were scored according to an established scale^7^ and the unused Nestlet material was weighed.

## Histology

### Histological validation of GFP expression

Upon completion of the behavioural tests, mice were transcardially perfused under deep, terminal anaesthesia induced by a mixture of ketamine and medetomidine (≥ 200 mg/kg ketamine, Zoetis, G; ≥ 2mg/kg medetomidine, Pfizer, US) injected i.p.. After perfusion with phosphate-buffered saline (PBS, Sigma, D) and, thereafter, 4 % paraformaldehyde (PFA) in PBS (SantaCruz, US), the brain was post-fixed in 4 % PFA/PBS for one night, followed by incubation in a storing solution (0.05 % sodium azide in PBS) at 4°C. Then, the PFC of the fixed brain was incubated for at least 1 h in a cutting solution (0.05 % sodium azide and 10 % sucrose in PBS) and cut into 60 µm coronal slices using a vibratome (VT1000, Leica, G) at room temperature (RT). Subsequently, the slices were transferred onto a multi-well plate filled with storing solution at 4°C. For inspection of GFP expression in the AAV-cohort, slices were washed with PBS (3x 10 min), stained with DAPI (0.0005 % w/v DAPI in PBS) for 20 min, washed again in PBS (3x 10 min), and mounted on glass slides in VectaShield (H-1000; Vector Labs, US) before imaging with an epifluorescence microscope (DM6, Leica).

### Immunohistochemistry (IHC)

To evaluate the ablation of GLUA1, tissue was used from 14 mice of the AAV-cohort transfected with either the GFP vector (7 mice) or GFP-Cre vector, as well as from 2-3 mice each of *Gria1*^ΔAmigo2^, *Gria1*^ΔGrik4^, and Cre-negative floxed-*Gria1* control mice of the transgenic cohorts (perfused and brains processed as described above). 60 µm slices were transferred into CELLSTAR (Cat# 662160 Greiner Bio-One, Austria) multi-well cell-culture plates and washed thrice for 10 min in PBS at room temperature (RT). They were thereafter immersed in a detergent (0.5% TritonX in PBS) for 20 min. To block potential nonspecific binding sites, the slices were kept in blocking solution containing 0.3% TritonX, 20 % normal goat serum (order# S-1000, Vector laboratories, USA) in PBS for 1 h at RT. After a short wash in PBS, the slices were then incubated over two nights at 4°C in carrier solution containing the primary antibody and 0.3% TritonX, 1 % normal goat serum in PBS. Polyclonal rabbit anti-GLUA1 (anti-GluR1; order# AB1504, Millipore, USA; diluted 1:500) was used as the primary antibody. After this incubation, slices were washed twice for 10 min in PBS at RT, and subsequently stained for 2 h in light-safe conditions at RT with a secondary AlexaFluor-546-labelled goat anti-rabbit antibody (order# A-11010, Invitrogen/ThermoFisher, USA) diluted at 1:1000 in carrier solution containing 0.05% TritonX, 2% normal goat serum in PBS. Slices were then briefly washed in PBS at RT, incubated for 10 min in a 1:1000 DAPI-solution diluted in PBS, and washed thrice for 10 min in PBS at RT. Finally, the slices were mounted on microscopy slides, they were embedded in VectaShield antifade mounting medium (VectorLabs) and stored at 4°C until epifluorescence (DM6, Leica; Aperio VERSA Brightfield, Fluorescence & FISH Digital Pathology Scanner, Leica) or confocal imaging (LSM 710, Leica).

# Supplementary Figures


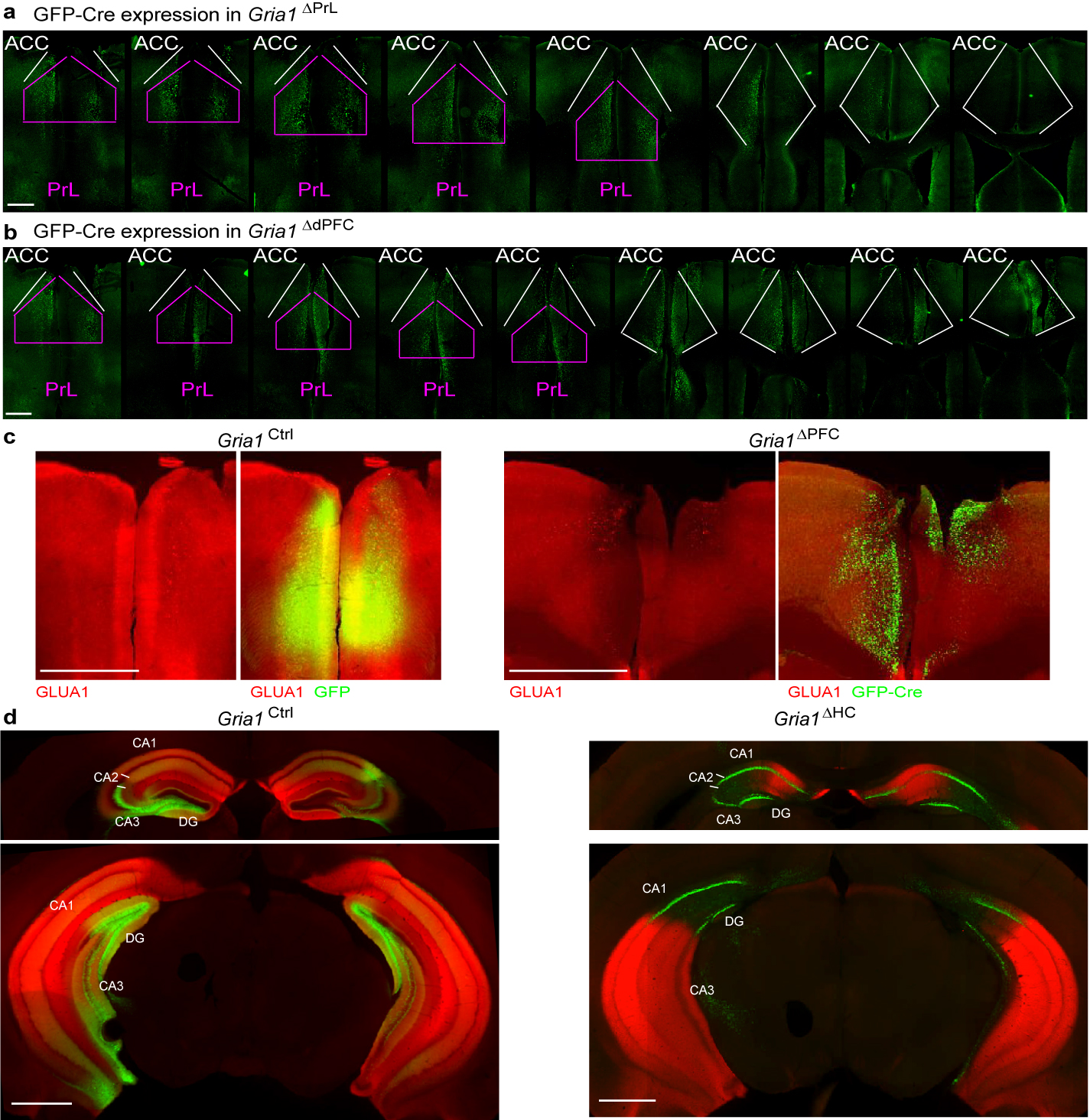


**Supplementary Figure 1.** **Ablation of GLUA1.** (**a-b**) Epifluorescence images of native GFP-Cre expression in mice transfected either specifically in PrL (a) or more broadly in dorsal PFC (b), see also main Fig. 1. (**c**) Epifluorescence images of anti-GLUA1 staining (red) and GFP-expression (green) in slices of PFC from floxed-*Gria1* mice infused either with a GFP-expressing control vector (left) or with a Cre-GFP into the PFC. The left image of each pair shows GLUA1 only whereas the right image shows an overlay of GLUA1 and GFP. (**d**) Epifluorescence images of anti-GLUA1 staining (red) and GFP-expression (green) in slices of dorsal (top) and ventral (bottom) hippocampus from floxed-*Gria1* mice infused either with a GFP-expressing control vector (left) or with a Cre-GFP into the hipocampus. Scale bar, 1 mm throughout.


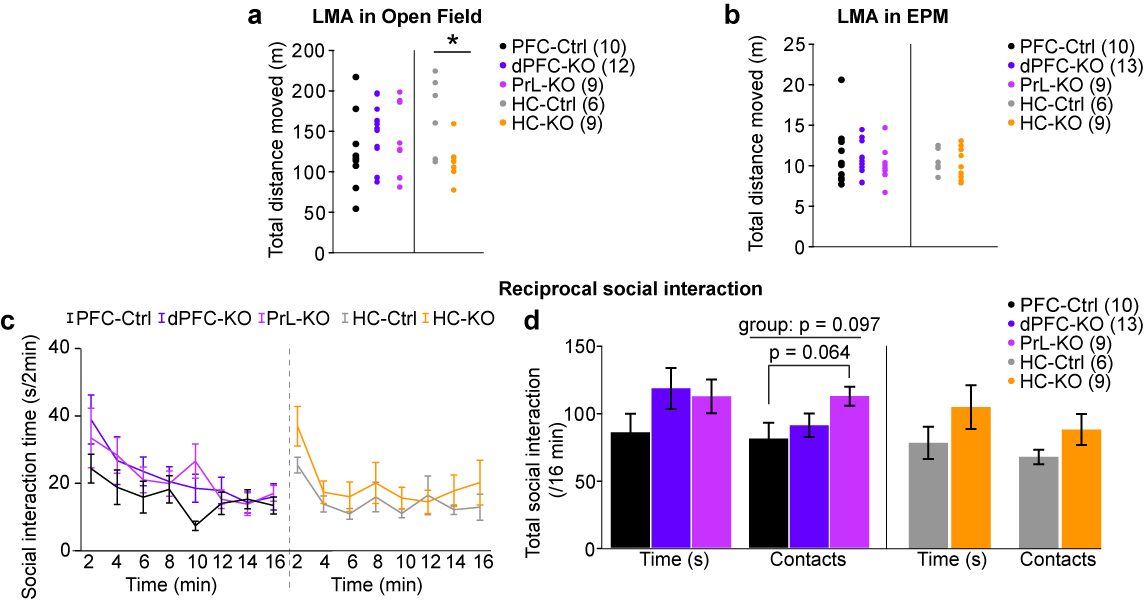


**Supplementary Figure 2.** **Ablation of GLUA1 prefrontal cortex and hippocampus affects locomotion and social interaction.** (**a**) Total distance moved in 90 min in a novel open field (a) or during 5 min in the elevated plus-maze (b), shown for individual mice of the groups identified by colour in (b). (**c**) Social interaction time in 2 min intervals in the cohorts identified in the legend for 16 min exposure. (**d**) Same as (b) but summed up total interaction time (left) and number of contacts (right). (c-d) shows mean±s.e.m. Data was analysed with ANOVA within the prefrontal and hippocampal cohorts, pairwise Dunnett post-hoc test was used in (d); see Supplementary Table 1-2 for statistical details and *N*-numbers. ^#^ *P* < 0.1, * *P* < 0.05.


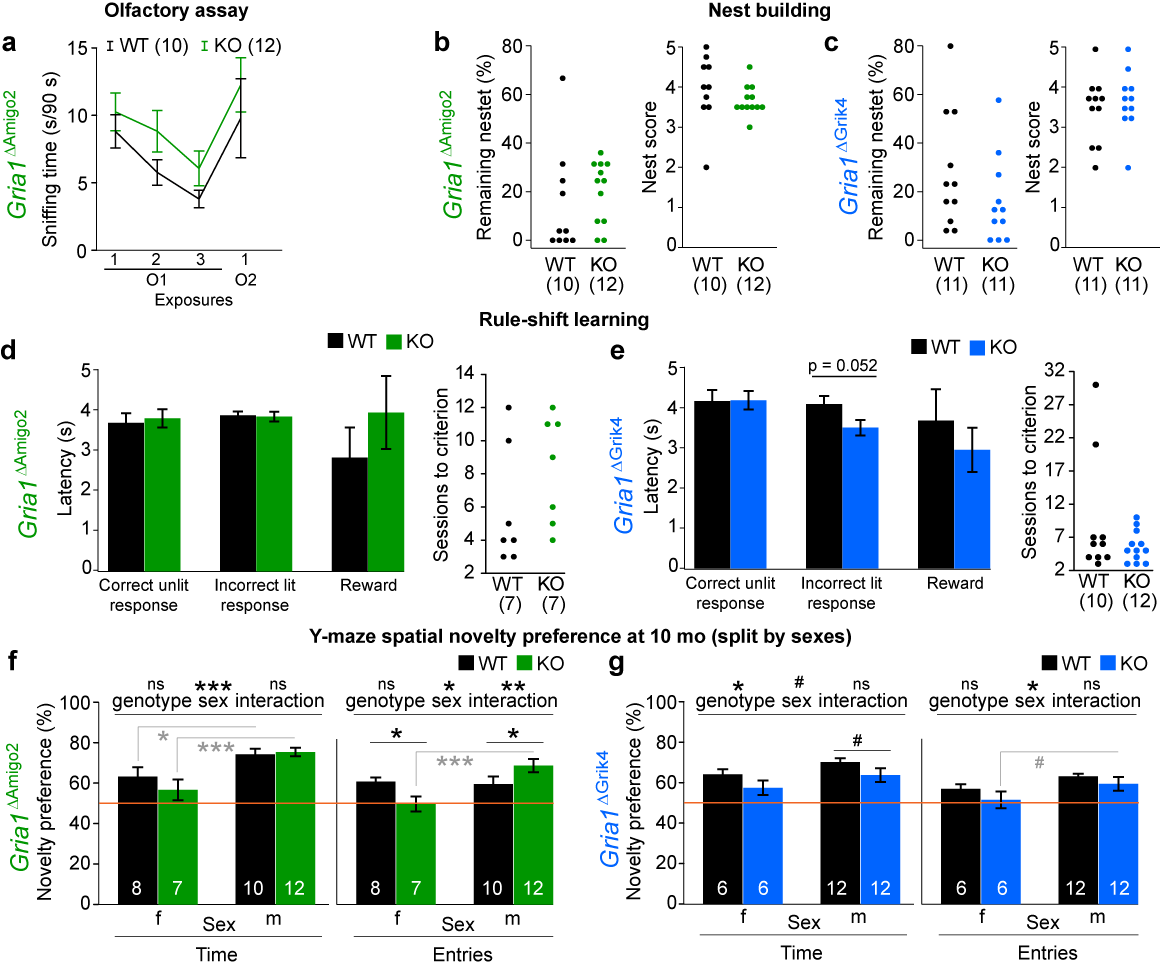


**Supplementary Figure 3.** **Effect of ablation of GLUA1 from CA2/CA3 on olfaction, nest-building, latencies during rule-shift and spatial-novelty preference.** (**a**) Time spent sniffing on cotton bud with either rose or lavender odour across 4 exposures of which the first three presented the same odour (O1). The test was only done in *Gria1*^ΔAmigo2^ mice because of their deficit in social memory. (**b,c**) Remaining nestlet (≥ 0.1 g, left) and nest score assigned to nest (right) after a night of nest building from pressed cotton pads (2.5-2.7 g) for the cohorts identified on the left. (**d,e**) Latencies for the actions identified on x-axes (left) and training sessions needed to achieve criterion (70% accuracy_unlit_ on two consecutive days, right) during rule-shift learning. (a, d-e) shows mean±s.e.m., (b-e) data from individual mice. (**f,g**) Novelty-preference data from the second test of spatial-novelty preference in the Y-maze (at the age of 10 mo), as displayed in main Figure 3c but broken down by sexes given that main effects of sex and a sex-genotype interaction (indicated above each panel) were found in this data. Data was analysed with univariate ANOVA and, where applicable, Sidak-adjusted pairwise post-hoc tests of simple main-effects within the indicated cohorts, see Supplementary Table 3-4 for statistical details. Grey indicators refer to Sidak-tests between sexes within the respective subgroups. *N*-numbers in brackets (a-e) or in the respective bars (f-g). ^ns^ *P* > 0.1, ^#^ *P* < 0.1, * *P* < 0.05, ** *P* < 0.01, *** *P* < 0.001.

# Supplementary Tables


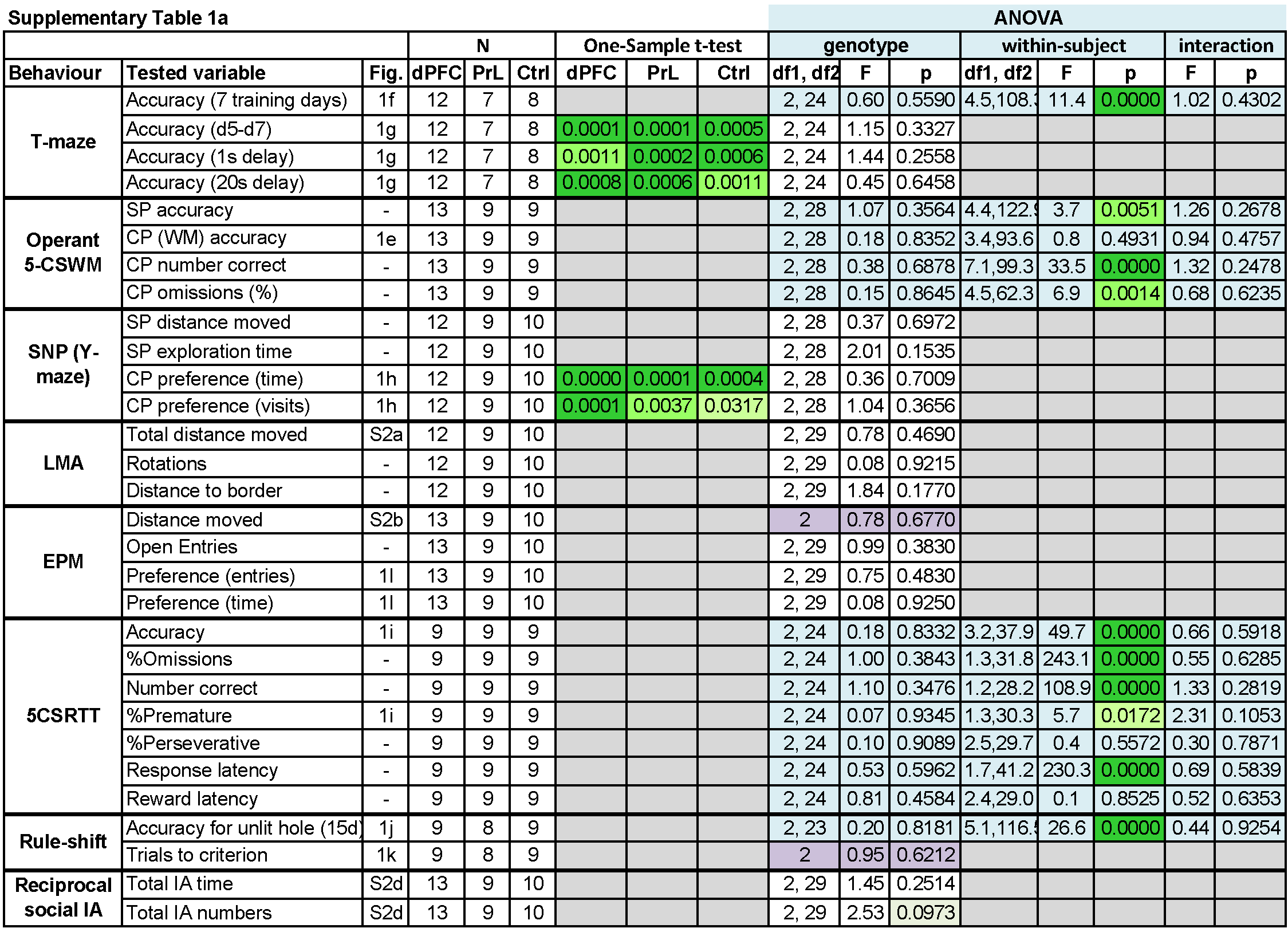


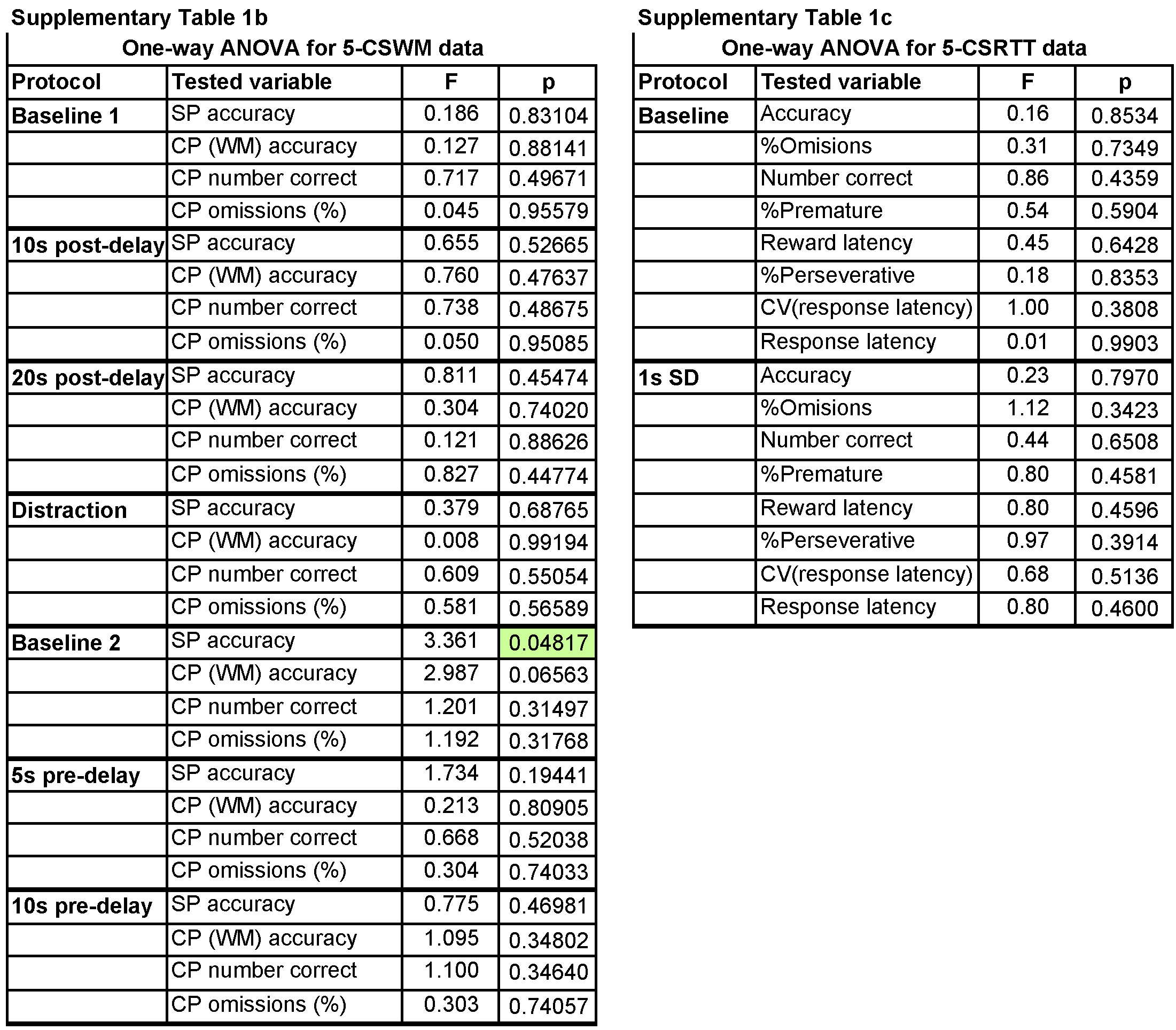


**Supplementary Table 1. Statistics of behavioural assessment after prefrontal GLUA1 ablation.** (**a**) Statistical assessment of behavior according to stated behavioural tests and variables (two left columns) using one-sample *t*-test against chance level where applicable and ANOVA. Use of repeated-measures (RM) ANOVA (Greenhouse-Geisser adjustment) to assess performance across multiple days or challenge conditions indicated in light blue. (**b-c**) Individual assessment of individual challenge conditions (left column) in the 5-CSWM (b) or the 5-CSRTT (c) regarding the named parameters using univariate ANOVA. In (a-c) *P*-values < .1 are indicated by shades of green; the lower the *P*-value, the darker. The number of animals contributing to each experiment per group are stated under “N” and the corresponding figure under “Fig.”. Numbers vary due to the exclusion of some animals that did not sufficiently acquire or participate in a task. The total number of animals in this cohort were 13 dPFC-KO, 9 PrL-KO, and 10 PFC-Ctrl mice. 3 further dPFC-KO mice had been excluded before analysis due to insufficient bilateral expression. Parameters analysed with non-parametric Kruskal-Wallis ANOVA are highlighted in purple, with stated numbers representing df, Kruskal-Wallis H (instead of *F*), and *P*-value. *Abbreviations:* CP, choice phase; LMA, novelty-induced locomotor activity; SNP, spatial-novelty preference; SP, sample phase.


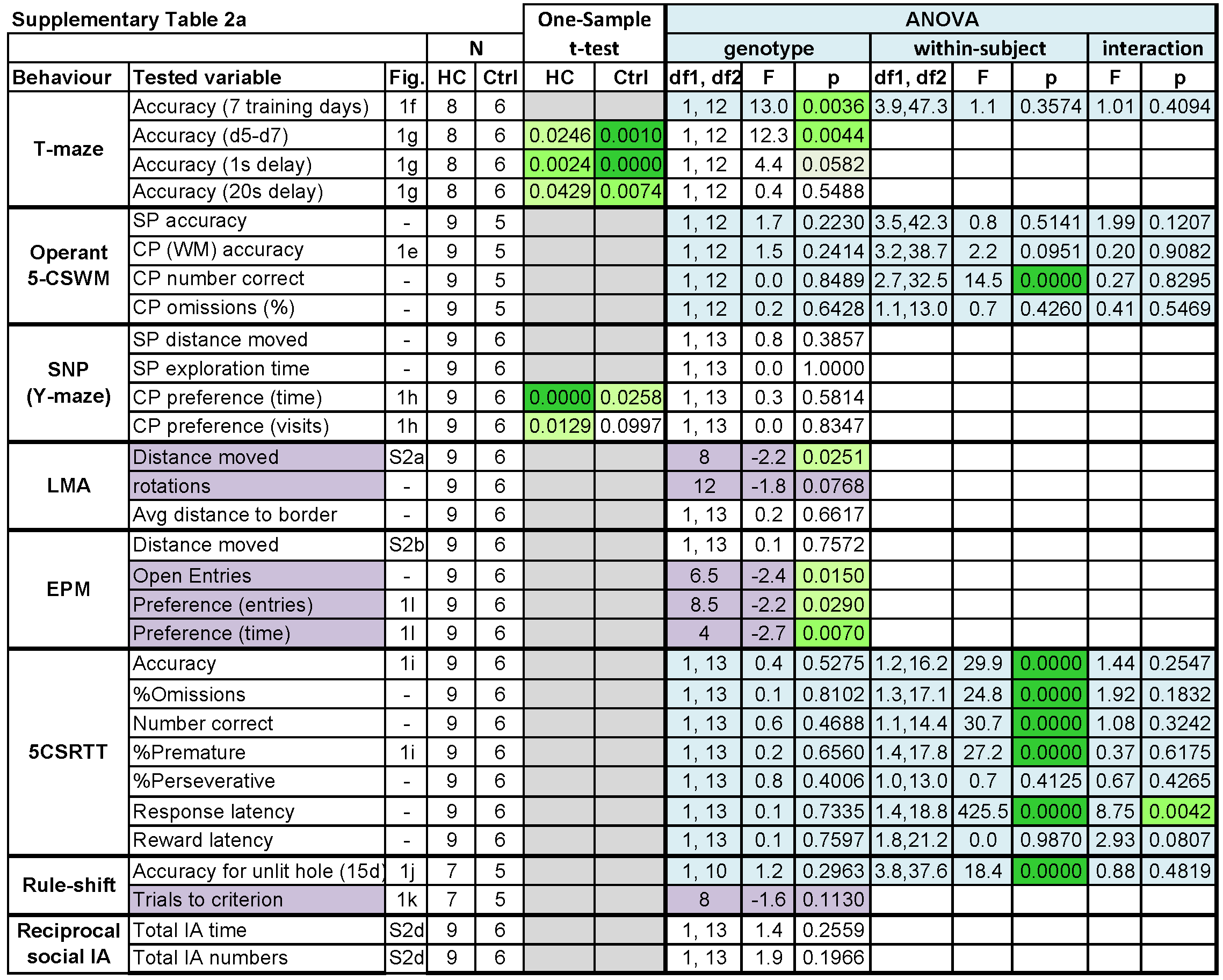


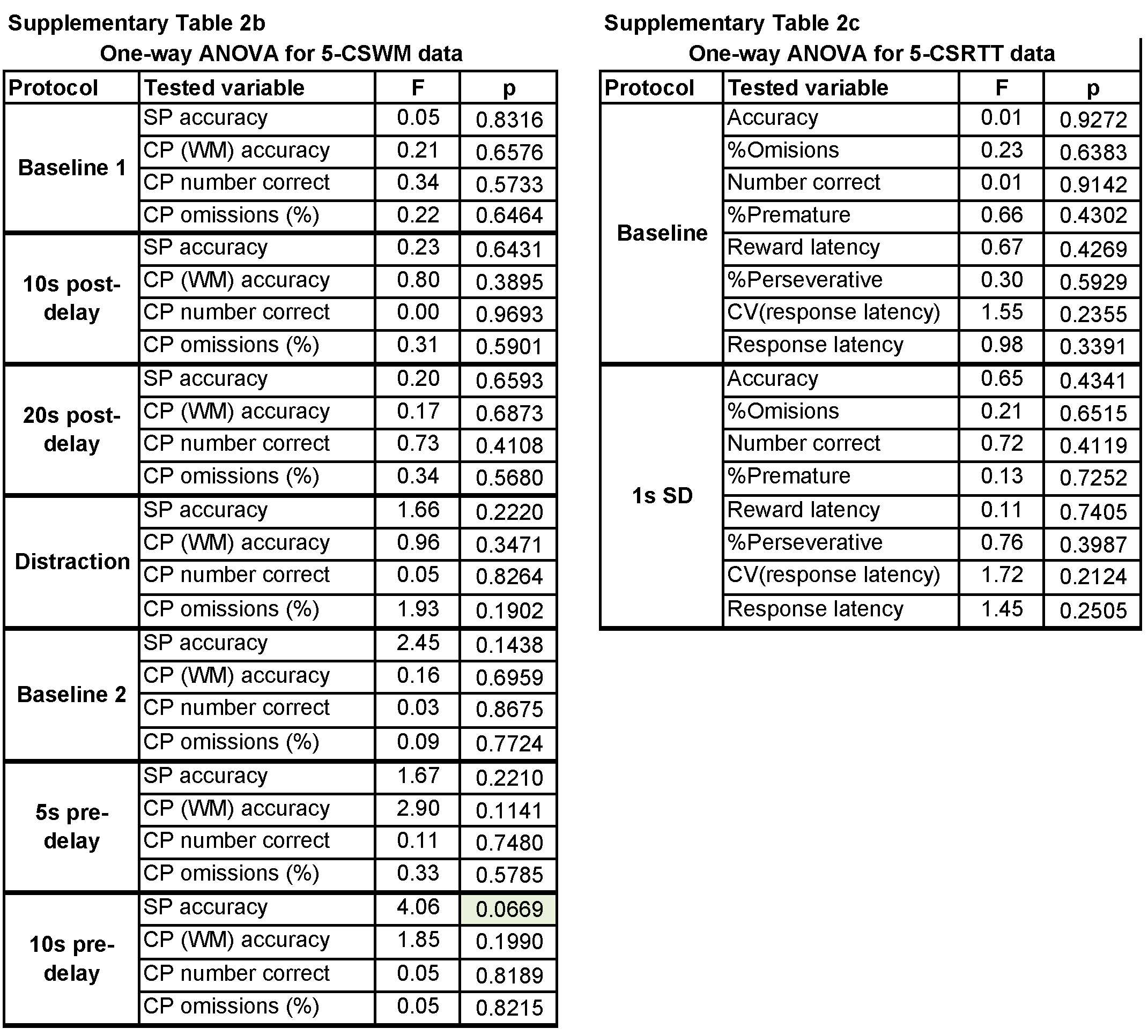


**Supplementary Table 2. Statistics of behavioural assessment after hippocampal GluA1 ablation.** Same as Supplementary Table 1 but for cohort with hippocampal transduction. (**a**) Statistical assessment of behavior according to stated tests and variables (two left columns) using one-sample *t*-test against chance level where applicable and ANOVA. Use of repeated-measures (RM) ANOVA (Greenhouse-Geisser adjustment) to assess performance across multiple days or challenge conditions indicated in light blue. Parameters analysed with non-parametric Mann-Whitney-U (MWU) test are highlighted in purple, with stated numbers representing MWU- (instead of df), Z- (instead of *F*) and *P*-values. (**b-c**) Individual assessment of individual challenge conditions (left column) in the 5-CSWM (b) or the 5-CSRTT (c) regarding the named parameters using univariate ANOVA. In (a-c) *P*-values < .1 are indicated by shades of green; the lower the *P*-value, the darker. Numbers vary due to the exclusion of some animals that did not sufficiently acquire or participate in a task. The total number of animals in this cohort were 9 HC-KO and 6 HC-Ctrl mice. 2 further HC-KO mice had been excluded before analysis due to insufficient bilateral expression. *Abbreviations:* CP, choice phase; LMA, novelty-induced locomotor activity; SNP, spatial-novelty preference; SP, sample phase.


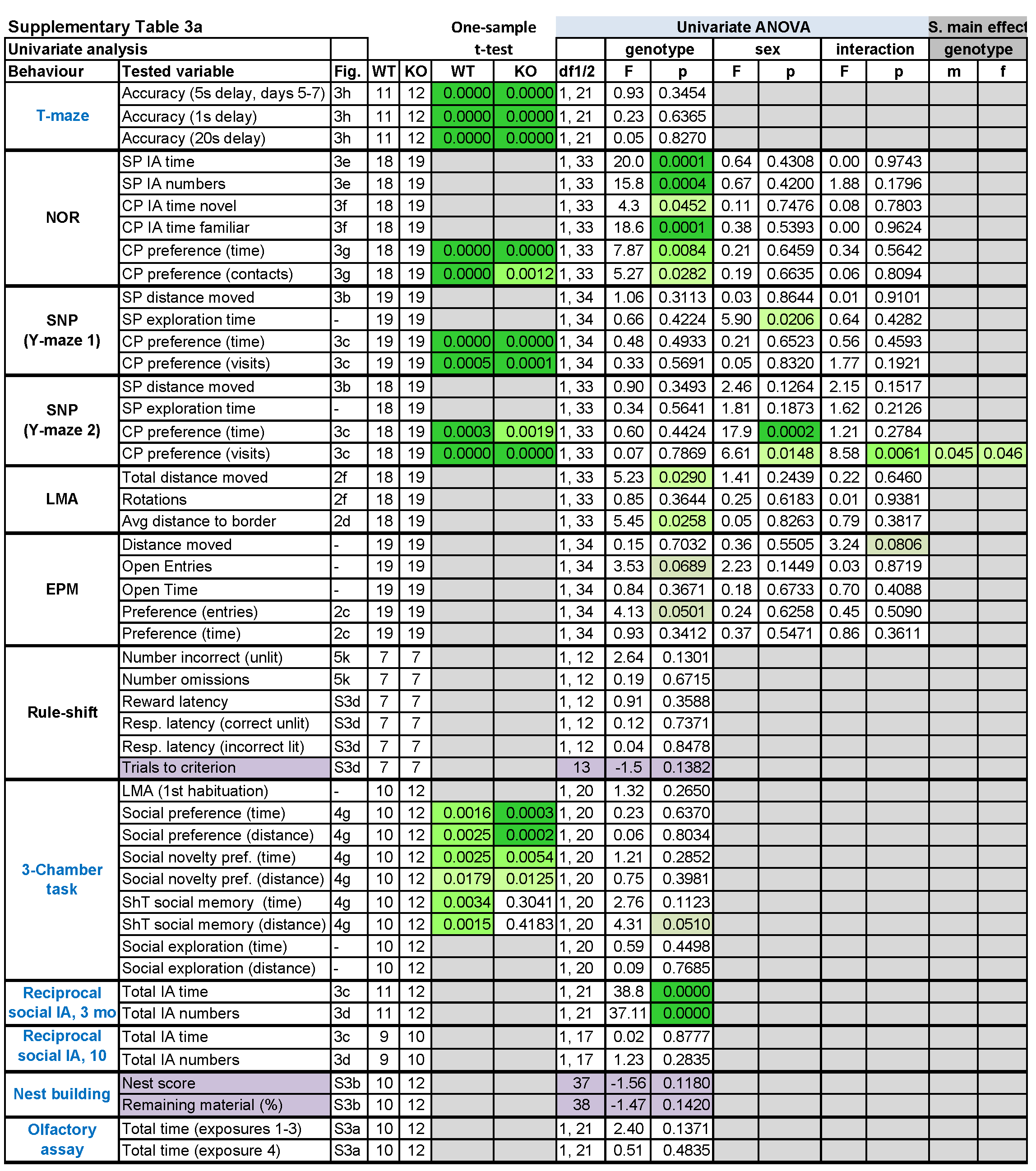


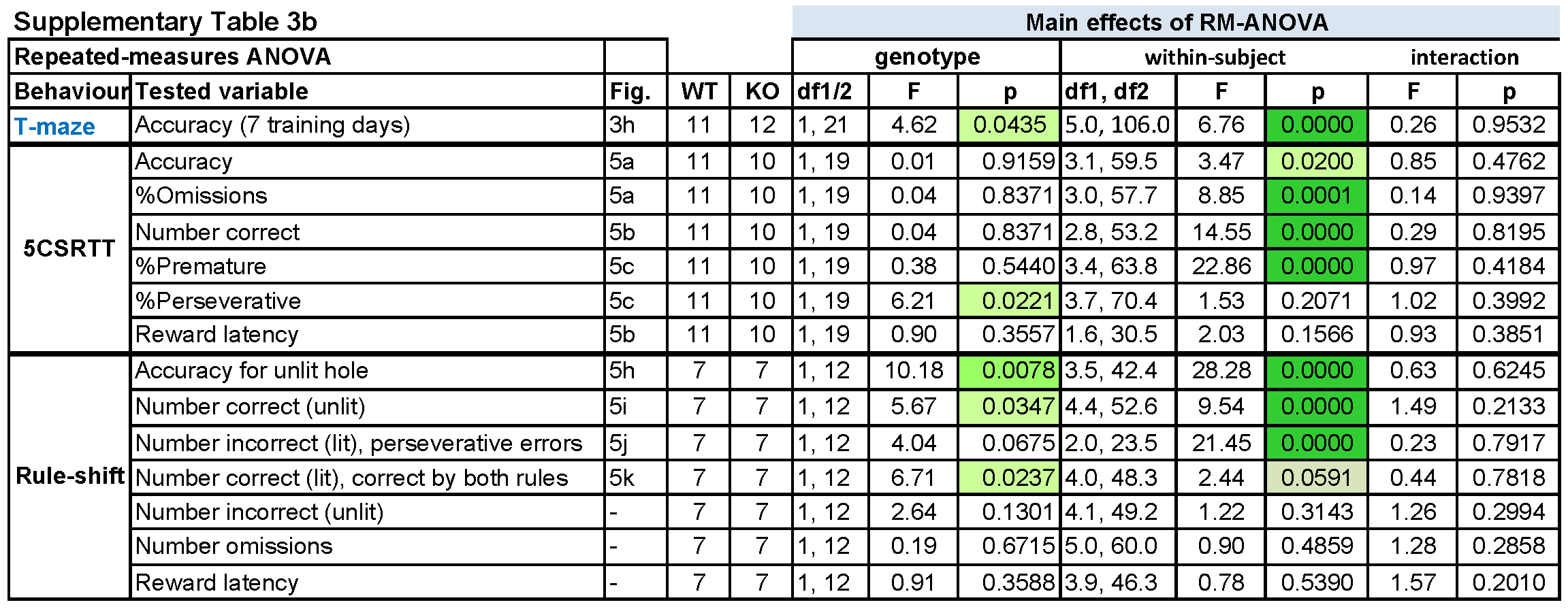


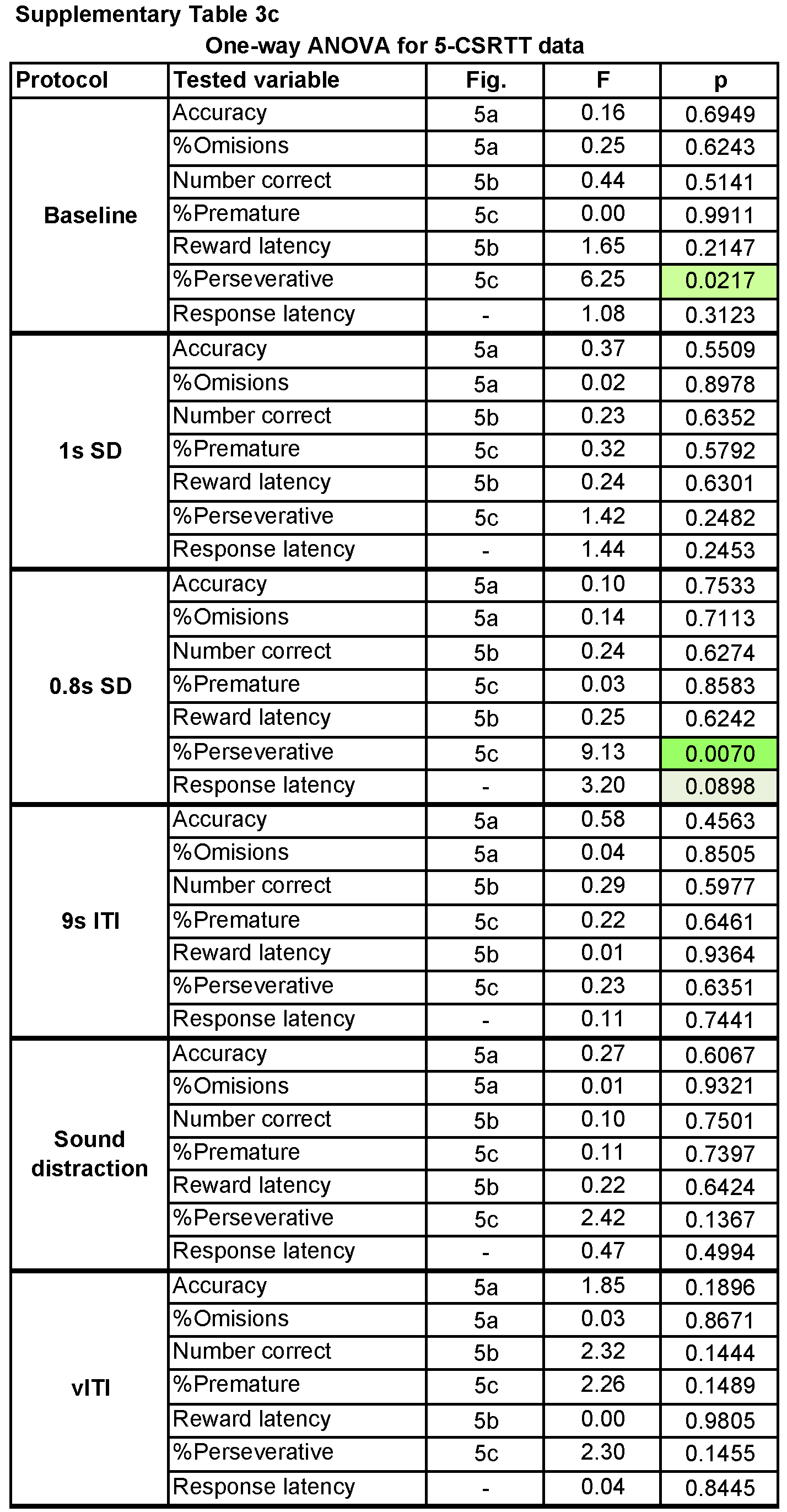


**Supplementary Table 3. Statistics of behavioural assessment after CA2-GLUA1 ablation.** (**a**) Statistical assessment of behavior according to stated behavioural tests and variables (two left columns) using one-sample *t*-test against chance level where applicable and univariate ANOVA. Sex was included as independent variable unless only the male part of the cohort was analysed (indicated by blue test name in first column) or the number of mice per genotype was considerably reduced because some animals did not reach training criterion. Parameters analysed with non-parametric Mann-Whitney-U (MWU) test are highlighted in purple, with stated numbers representing MWU- (instead of df), Z- (instead of *F*) and *P*-values. (**b**) Assessment of performance across multiple days or challenge conditions using repeated-measures (RM) ANOVA (Greenhouse-Geisser adjustment). (**c**) Statistical assessment of individual challenge conditions (left column) in the 5-CSRTT regarding the named parameters using univariate ANOVA. In (a-c) *P*-values < .1 are indicated by shades of green; the lower the *P*-value, the darker. The number of animals contributing to each experiment per group are stated under “N” and the corresponding figure under “Fig.”. Numbers vary due to the exclusion of some animals that did not sufficiently acquire or participate in a task. The total number of animals in this cohort were 19 *Gria1*^Ctrl^ (11 male, 8 female) and 19 *Gria1*^ΔAmigo2^ (12 male, 7 female) mice. Tests stated in blue font were conducted only in males. *Abbreviations:* CP, choice phase; IA, interaction; LMA, novelty-induced locomotor activity; SNP, spatial-novelty preference; SP, sample phase.


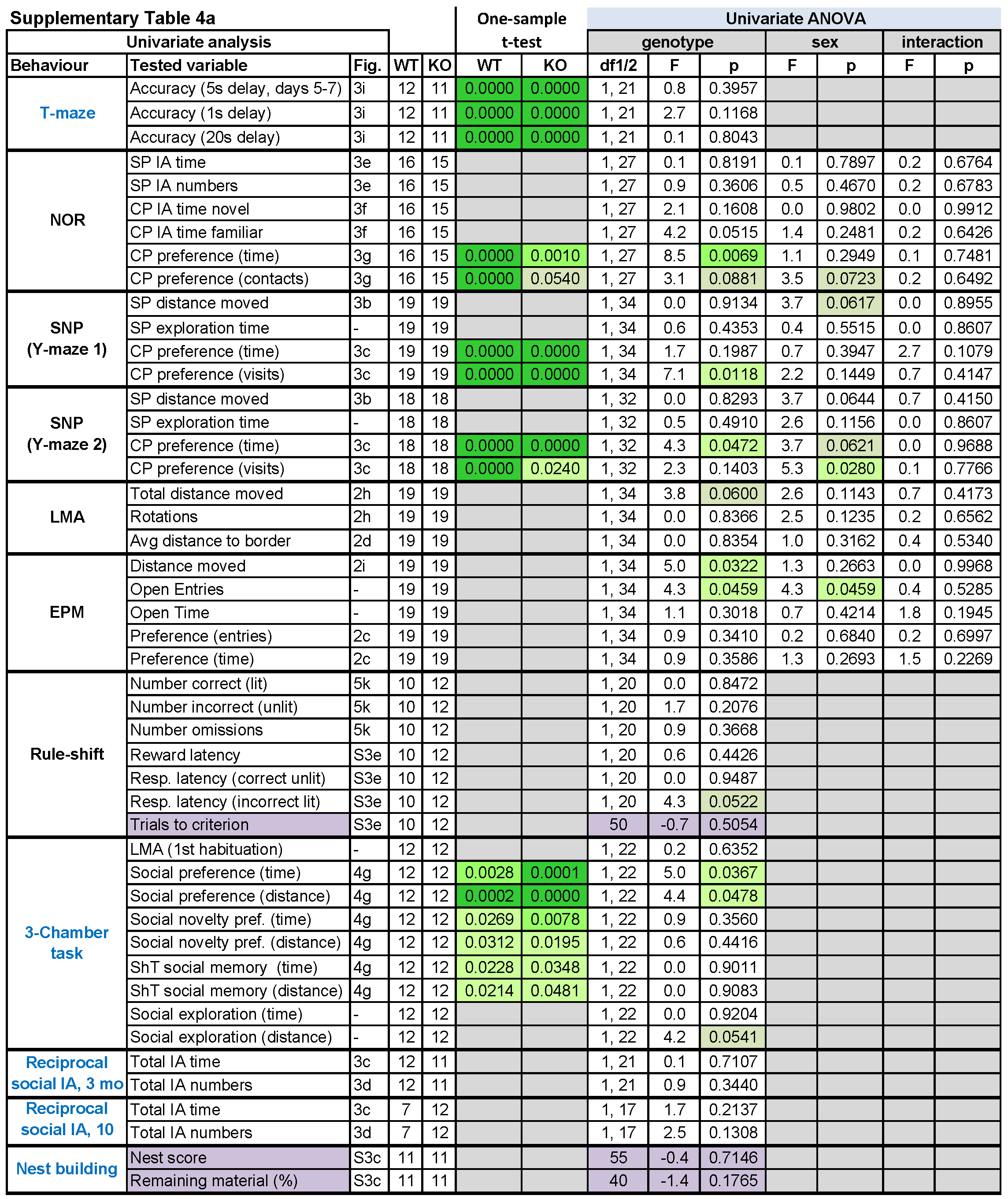


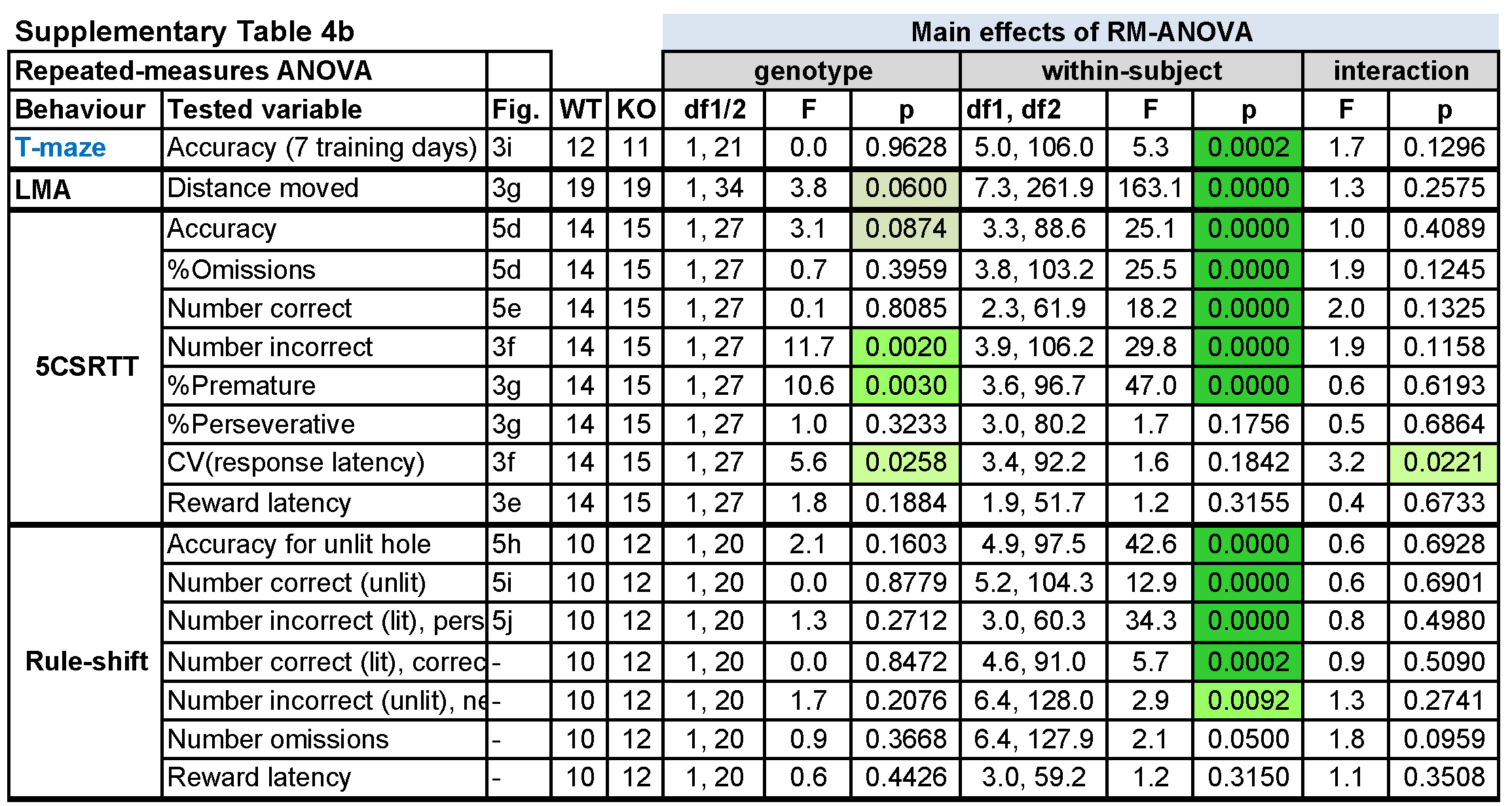


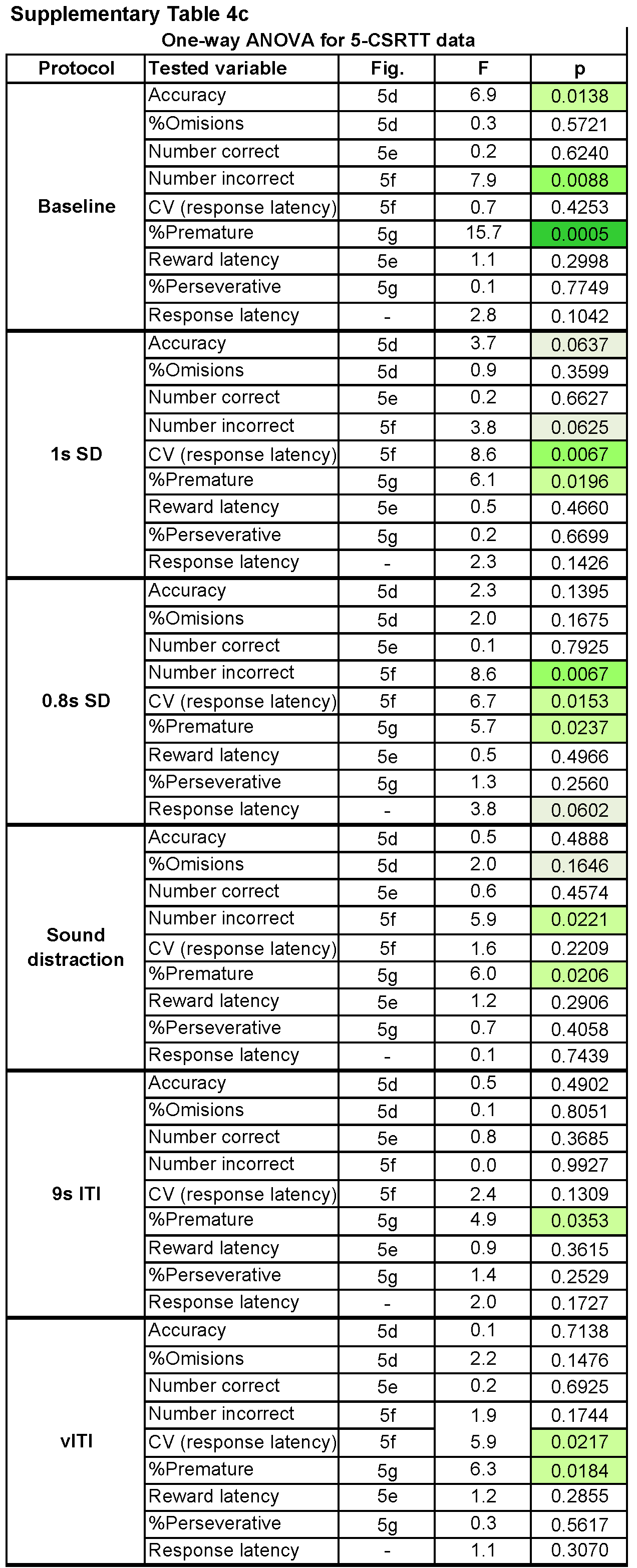


**Supplementary Table 4. Statistics of behavioural assessment after CA3-GLUA1 ablation.** Similar to Supplementary Table 3, but for *Gria1*^ΔGrik4^ cohort. (**a**) Statistical assessment of behavior according to stated behavioural tests and variables (two left columns) using one-sample *t*-test against chance level where applicable and univariate ANOVA. Sex was included as independent variable unless only the male part of the cohort was analysed (indicated by blue test name in first column) or the number of mice per genotype was considerably reduced because some animals did not reach training criterion. Parameters analysed with non-parametric Mann-Whitney-U (MWU) test are highlighted in purple, with stated numbers representing MWU- (instead of df), Z- (instead of *F*) and *P*-values. (**b**) Assessment of performance across multiple days or challenge conditions using repeated-measures (RM) ANOVA (Greenhouse-Geisser adjustment). (**c**) Statistical assessment of individual challenge conditions (left column) in the 5-CSRTT regarding the named parameters using univariate ANOVA. In (a-c) *P*-values < .1 are indicated by shades of green; the lower the *P*-value, the darker. The number of animals contributing to each experiment per group are stated under “N” and the corresponding figure under “Fig.”. Numbers vary due to the exclusion of some animals that did not sufficiently acquire or participate in a task. The total number of animals in this cohort were 19 *Gria1*^Ctrl^ (12 male, 7 female) and 19 *Gria1*^ΔGrik4^ (12 male, 7 female) mice. Tests stated in blue font were conducted only in males. *Abbreviations:* CP, choice phase; IA, interaction; LMA, novelty-induced locomotor activity; SNP, spatial-novelty preference; SP, sample phase.

|  | **Parameters of training and baseline stages** | | | | | | | | |
| --- | --- | --- | --- | --- | --- | --- | --- | --- | --- |
| Stage | SP-SD, s | | CP-SD, s | Pre-delay, s | | Post-delay, s | | Reward (μl) | CP configurations |
| 1 | 20 | | 20 | 0 | | 2 | | 20 / 10 / 0 | 1-4, 2-5 |
| 2 | 20 | | 20 | 0 | | 2 | | 10 / 0 | 1-3, 2-4, 3-5 |
| 3 | 20 | | 20 | 0 | | 2 | | 10 | 1-2, 1-3, 1-4, 2-3, 2-4, 2-5, 3-4, 3-5, 4-5 |
| 4 | 8 | | 20 | 0 | | 2 | | 10 | 1-2, 1-3, 1-4, 2-3, 2-4, 2-5, 3-4, 3-5, 4-5 |
| 5, BL1 | 8 | | 20 | 0 | | 2 | | 0 | 1-2, 1-3, 1-4, 2-3, 2-4, 2-5, 3-4, 3-5, 4-5 |
| 6, BL2 (pre-delay) | 8 | | 20 | 0 | | 2 | | 10 | 1-2, 1-3, 1-4, 2-3, 2-4, 2-5, 3-4, 3-5, 4-5 |
|  | **Parameters of challenge stages** | | | | | | | | |
| Pre-Delay 5s | 8 | 20 | | 5 | 2 | | 10 | | As baseline |
| Pre-Delay 10s | 8 | 20 | | 10 | 2 | | 10 | | As baseline |
| Distraction | 8 | 20 | | 0 | 5 (Distract.) | | 0 | | As baseline |
| Post-Delay 10s | 8 | 20 | | 0 | 12 (2+10) | | 0 | | As baseline |
| Post-Delay 20s | 8 | 20 | | 0 | 22 (2+20) | | 0 | | As baseline |

**Supplementary Table 5. Training and test stages of the 5-CSWM task.** The reward volume relates to the SP. CP reward was always 60 μl. The SP reward was decreased as mice reached ≥25 correct SP responses on three consecutive days within each stage. The mice transitioned incrementally to the next main training stage (from 1 through to 5) if they achieved ≥70% CP accuracy_lit_ and ≥10 correct CP responses in 30 min in three consecutive sessions. Additionally, to transition from stage 3 to 4 (reduction of SP-SD) mice had to achieve an SP accuracy of ≥80% in three consecutive sessions. To transition from stage 5 (baseline 1) to the first challenge protocol, mice had to achieve at least ≥60% CP accuracy_lit_ and ≥9 correct CP responses in 30 min on stage 5. BL1, baseline stage 1, use before challenges with post-delay extension or distraction; BL2, baseline 2, used before challenges with pre-delay extension. CP-SD, stimulus duration in the choice phase; SP-SD, stimulus duration in the sample phase.

|  | **Parameters of training** | | | | **Criteria for stage transition (2 consecutive days)** | | | |
| --- | --- | --- | --- | --- | --- | --- | --- | --- |
| **Stage** | SD (s) | | LH (s) | ITI (s) | # correct | % correct | % accuracy | %omissions |
| **1** | 20 | | 22 | 2 | >= 30 | >= 40 | - | - |
| **2** | 8 | | 10 | 2 | >= 40 | >= 50 | - | - |
| **3** | 8 | | 10 | 5 |  |  | >= 80 | <= 50 |
| **4** | 4 | | 6 | 5 |  |  | >= 80 | <= 50 |
| **5, BL** | 2 | | 4 | 5 |  |  | >= 80 | <= 50 |
| **Challenge** | | | | | | | | |
| 6 | | 1 | 3 | 5 | Attention challenge, 1s SD | | | |
| 7 | | 0.8 | 2.8 | 5 | Attention challenge, 0.8 SD | | | |
| 8 | | 2 | 4 | 5 | Auditory distraction: 1s white-noise of 70dB randomly between 0.5-3.5 s of the ITI | | | |
| 9 | | 2 | 4 | 9 | 9s fixed ITI impulsivity challenge | | | |
| 10 | | 2 | 4 | 7, 9, 11, 13 | Variable ITI impulsivity challenge | | | |

**Supplementary Table 6. Training and test stages of the 5-CSRTT.** The limited hold (LH) period includes the SD. Stage 4 was the baseline of the AAV-cohort.

# Supplementary references

1 Kapanaiah SKT, van der Veen B, Strahnen D, Akam T, Kätzel D. A low-cost open-source 5-choice operant box system optimized for electrophysiology and optophysiology in mice. *Sci Rep* 2021; **11**: 22279.

2 Akam T, Lustig A, Rowland JM, Kapanaiah SK, Esteve-Agraz J, Panniello M *et al.* Open-source, Python-based, hardware and software for controlling behavioural neuroscience experiments. *eLife* 2022; **11**: e67846.

3 Grimm CM, Aksamaz S, Schulz S, Teutsch J, Sicinski P, Liss B *et al.* Schizophrenia-related cognitive dysfunction in the Cyclin-D2 knockout mouse model of ventral hippocampal hyperactivity. *Transl Psychiatry* 2018; **8**: 212.

4 Bari A, Dalley JW, Robbins TW. The application of the 5-choice serial reaction time task for the assessment of visual attentional processes and impulse control in rats. *Nat Protoc* 2008; **3**: 759–767.

5 Kilonzo K, Veen B van der, Teutsch J, Schulz S, Kapanaiah SKT, Liss B *et al.* Delayed-matching-to-position working memory in mice relies on NMDA-receptors in prefrontal pyramidal cells. *Sci Rep* 2021; **11**: 1–19.

6 Hitti FL, Siegelbaum SA. The hippocampal CA2 region is essential for social memory. *Nature* 2014; **508**: 88–92.

7 Deacon RM. Assessing nest building in mice. *Nat Protoc* 2006; **1**: 1117–1119.

.
